# Amorphous Solid Dispersions of Polyphenols: Current State of the Art (Part I)

**DOI:** 10.3390/ph19040598

**Published:** 2026-04-08

**Authors:** Natalia Rosiak, Miłosz Ignacyk, Aleksandra Kryszak, Jakub Piontek, Judyta Cielecka-Piontek

**Affiliations:** 1 Department of Pharmacognosy and Biomaterials, Faculty of Pharmacy, Poznan University of Medical Sciences, 3 Rokietnicka St., 60-806 Poznan, Poland; nrosiak@ump.edu.pl (N.R.); milosz.ignacyk@student.ump.edu.pl (M.I.);; 2 Department of Occupational Therapy, Poznan University of Medical Sciences, Swięcickiego 6, 60-781 Poznan, Poland

**Keywords:** amorphous solid dispersion, co-amorphous solid dispersion, polyphenols, hot-melt extrusion, spray drying, amorphization

## Abstract

Polyphenols have attracted considerable scientific interest over recent years due to their broad spectrum of biological activities, including antioxidant, cardioprotective, anti-inflammatory, antidiabetic, and anticancer properties. However, their practical application is often limited by unfavorable physicochemical characteristics, particularly low aqueous solubility. Consequently, amorphous solid dispersions (ASDs) have been extensively investigated as a formulation strategy to overcome these limitations. This article represents the first part of a two-part review and presents the current state of the art in amorphous solid dispersions of polyphenols. The available literature is systematically summarized with respect to the investigated polyphenolic compounds, the employed carriers (with particular emphasis on polymeric systems), the preparation methods, and the solid-state characterization techniques used to confirm amorphization. Both single-component systems and binary combinations of polyphenols reported in the literature are considered. The collected data are presented in tabular form and complemented by a heat map illustrating the frequency of reported polyphenol–carrier combinations. The aim of this review is to organize the available knowledge, identify the most extensively studied systems, and highlight research areas that remain underexplored. A detailed discussion of the pharmaceutical benefits and mechanistic aspects of polyphenols in ASD systems will be provided in Part II.

## 1. Introduction

**Figure 1:**
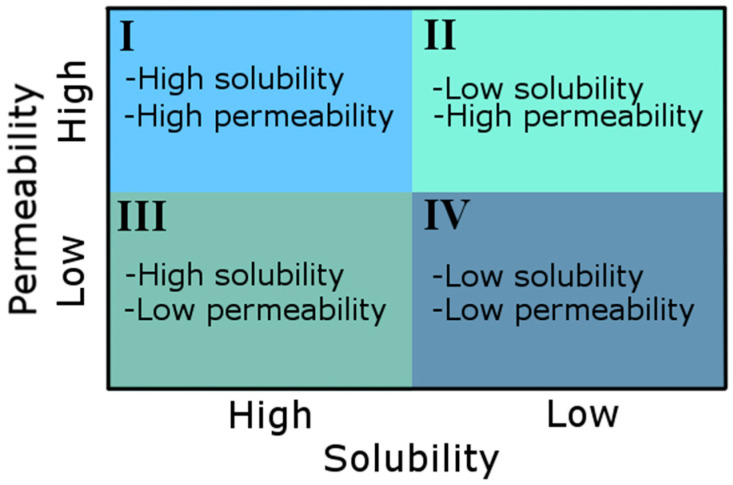
Biopharmaceutical classification system (BCS).

Polyphenols are a diverse group of plant-derived bioactive compounds that are widely present in the human diet and have been associated with numerous health-promoting effects, including antioxidant [1,2,3,4], antidiabetic [5,6], anti-inflammatory [7,8], anticancer [9,10], cardioprotective [11], and neuroprotective [8,12] activities. Despite these promising biological properties, the practical application of polyphenols in functional foods and pharmaceutical formulations is often limited by their low oral bioavailability. The low bioavailability of many polyphenols primarily results from their poor aqueous solubility, which restricts dissolution and absorption in the gastrointestinal tract and consequently limits the attainment of therapeutically relevant concentrations in vivo [13,14,15]. According to the biopharmaceutical classification system (BCS), many polyphenols fall into classes characterized by low solubility, where improving dissolution behavior may directly enhance their permeability and overall bioavailability (Figure 1) [16].

**Figure 2:**
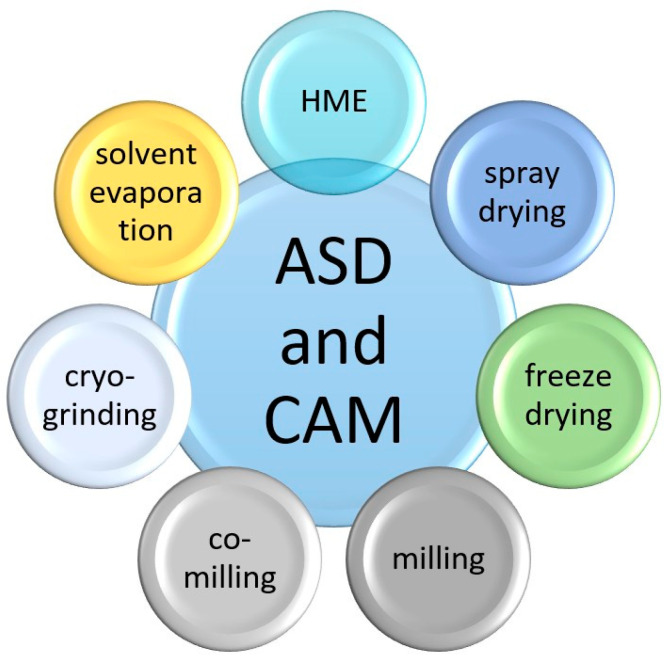
Techniques used in the process of obtaining amorphous solid dispersion (ASDs) and co-amorphous solid dispersions (CAMs).

To overcome these limitations, various formulation strategies, such as the use of cocrystals, liposomes, inclusion complexes, and nanofibers, have been explored to enhance the solubility and biological performance of polyphenols [17]. Among these approaches, the use of amorphous solid dispersions (ASDs) or co-amorphous solid dispersions (CAMs) has emerged as a particularly promising formulation strategy. ASDs are systems in which a poorly water-soluble compound, such as a polyphenol, is molecularly dispersed within a solid carrier matrix typically composed of hydrophilic polymers [18,19]. In contrast, CAMs are formed by combining the drug with one or more low-molecular-weight co-formers (e.g., amino acids, organic acids, or other small molecules) to create a homogeneous amorphous phase stabilized by intermolecular interactions [20,21,22,23,24,25,26,27,28]. The conversion of polyphenols into an amorphous state offers an increased apparent solubility and dissolution rate due to the higher free energy of the disordered solid form. However, the inherent thermodynamic instability of amorphous systems necessitates appropriate stabilization strategies, typically achieved through intermolecular interactions with a carrier matrix or co-former molecules that inhibit recrystallization. Numerous processing techniques have been developed to obtain stable ASDs and CAMs, many of which are already well established in the pharmaceutical and food industries (Figure 2) [29,30,31,32,33,34,35]. Polyphenols represent a pharmacologically relevant yet formulation-challenging subclass of poorly soluble bioactive compounds, which justifies a focused analysis of ASD strategies in this group.

This review is divided into two parts. Part I focuses on the formulation design of ASDs and CAMs, providing an overview of the investigated compounds, the selection of polymeric carriers or co-formers, and the preparation techniques used to obtain amorphous systems. Particular attention is given to experimental design considerations, including factors that should be taken into account when selecting formulation components and processing methods. This section also discusses the analytical techniques used to confirm the amorphous state and to identify polyphenol–polymer or polyphenol–co-former interactions, as well as their role in stabilizing the amorphous phase and preventing recrystallization. Part II will address the biopharmaceutical performance of these systems, including the influence of carrier selection on the release profile of polyphenols from the matrix; pH-dependent dissolution behavior; and the outcomes of in vitro and in vivo studies evaluating improvements in solubility, bioavailability, and biological activity.

## 2. Scope and Methodology of the Review

This review focuses on ASDs and co-amorphous systems of polyphenolic compounds, including flavonoids, stilbenes, phenolic acids, and related derivatives, developed to improve physicochemical properties, particularly solubility, dissolution behavior, physical stability, and oral bioavailability. Both binary and multicomponent systems incorporating carriers, low-molecular-weight co-formers, or combinations thereof were considered.

### 2.1. Time Frame

According to data available in the PubMed database (www.pubmed.ncbi.nlm.nih.gov), the first reports on amorphous solid dispersions of polyphenols date back to 2004. Therefore, the temporal scope of this review was defined to encompass 2004 to the end of December 2025.

### 2.2. Search Strategy and Keywords

The literature search was performed across multiple databases, including PubMed, Scopus, Web of Science, and Google Scholar, using combinations of keywords such as: amorphous solid dispersion, co-amorphous, ASD, ASD of polyphenols, amorphous solid dispersion of polyphenols, polyphenol, flavonoid, phenolic compound, hot-melt extrusion, spray drying, supercritical fluid, mechanochemical processing, solvent evaporation, ASD solvent evaporation, glass transition temperature, and effect halo.

Additionally, compound-specific searches were performed using the following formula: ASD of (compound) and amorphous solid dispersion of (compound), where (compound) was replaced with the following substances: apigenin, baicalein, baicalin, butein, chrysin, curcumin, daidzein, diosmin, diosmetin, ellagic acid, epigallocatechin gallate, ferulic acid, fisetin, genistein, hesperidin, hesperetin, kaempferol, luteolin, magnolol, myricetin, myricitrin, naringenin, naringin, nobiletin, oxyresveratrol, pterostilbene, polydatin, quercetin, resveratrol, rutin, sinapic acid, and wogonin.

Relevant publications were identified through manual screening of the reference lists of selected articles.

A patent literature search was performed using the Lens.org and Google Patents databases. The search was limited to patent applications and granted patents within the US, EU, and WO-WIPO jurisdictions and restricted to documents classified under CPC code A61K9/146.

### 2.3. Inclusion Criteria

Studies were included in this review if they met the following criteria:

- The investigated compound belonged to the group of polyphenols, including flavonoids, stilbenes, phenolic acids, or their derivatives;
- The formulation involved an amorphous solid dispersion, co-amorphous system, or amorphous multicomponent system;
- The amorphous nature of the system was experimentally confirmed using solid-state characterization techniques (e.g., DSC, XRPD);
- The study reported physicochemical, biopharmaceutical, or biological performance metrics of the amorphous system, such as solubility, dissolution behavior, stability, permeability, or bioavailability;
- The article or patent was published in a peer-reviewed scientific journal within the defined time frame (2004–December 2025).

### 2.4. Exclusion Criteria

Studies were excluded from the review if they met any of the following criteria:

- The formulation did not involve an amorphous solid dispersion or co-amorphous system;
- The investigated compound was not a polyphenol;
- The amorphous character of the system was not experimentally verified
- The study focused exclusively on crystalline forms, emulsions, inclusion complexes, liposomes, or nanocrystals without an amorphous phase;
- The publication was a conference abstract, editorial, or commentary without original experimental data;
- Duplicate records;
- Studies with insufficient methodological detail.

The review includes original research articles reporting the preparation, characterization, and performance of amorphous polyphenol-based systems. Review articles were used selectively for background information and contextualization. Studies not involving amorphous systems or not focused on polyphenolic compounds were excluded.

## 3. Polyphenols Investigated in Amorphous Solid Dispersions

The literature reports numerous attempts to employ ASDs to improve the physicochemical properties of polyphenols, which are often characterized by low aqueous solubility and limited bioavailability. Studies on ASDs have investigated both individual polyphenolic compounds and binary systems comprising combinations of bioactive substances. The analyzed polyphenols represent a broad range of chemical classes (including flavones, flavonols, flavanones, stilbenes, and phenolic acid derivatives), reflecting sustained research interest in these compounds in the context of amorphous formulations. This section summarizes the polyphenols that have been investigated in amorphous solid dispersion systems, providing an overview of their prevalence in the literature and general research trends.

### 3.1. Literature Overview

An overview of ASDs of polyphenols reported in the literature, including the investigated compounds, carriers, preparation methods, and solid-state characterization techniques, is summarized in Table 1.

**Figure 3:**
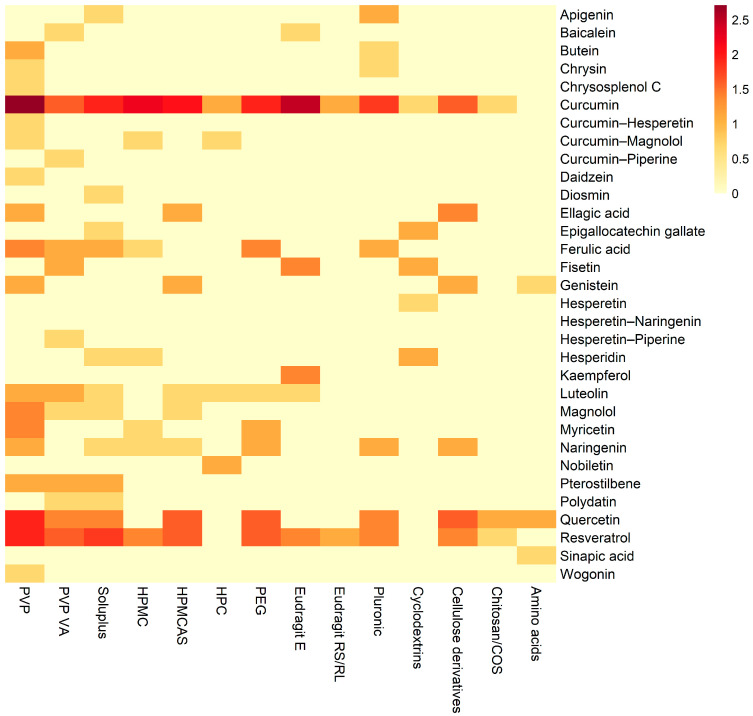
Heat map of reported amorphous solid dispersions of polyphenols with various carriers. Color intensity indicates the number of publications.

To complement the tabulated overview and facilitate visual comparison, the distribution of reported amorphous solid dispersion systems is summarized using a heat map. Figure 3 presents a heat map illustrating the distribution of amorphous solid dispersion systems across individual polyphenols and carriers.

The most documented studies concern curcumin, quercetin, and resveratrol, which have been investigated in combination with a wide variety of polymeric carriers. In contrast, a substantial proportion of polyphenols has been addressed in only a limited number of reports, often restricted to a single carrier or a single study. Regarding polymeric carriers, the most commonly used polymers include polyvinylpyrrolidone-based polymers (PVP and PVP VA), cellulose derivatives (HPMC and HPMCAS), polyethylene glycol, and methacrylate polymers from the Eudragit group. These carriers appear in numerous combinations with different polyphenols, indicating their widespread application in ASD research.

The studies summarized in Table 1 are further discussed below, with emphasis on the formulation design stage of ASDs. Particular attention is given to the composition of the systems, preparation methods, and the analytical techniques employed to confirm amorphization and investigate drug–polymer interactions. Such physicochemical characterization is fundamental for understanding the structural properties and stability of ASDs, which ultimately determine their performance in improving solubility, bioavailability, and biological activity.

**Apigenin (APG)**

Stasiłowicz-Krzemień et al. [36] developed an amorphous solid dispersion of APG with Soluplus (SOL) using supercritical carbon dioxide processing. XRPD analysis confirmed the complete amorphization of APG in the APG-SOL system, as evidenced by the disappearance of characteristic Bragg reflections and the presence of a broad amorphous halo. **FT-IR** indicated the formation of stabilizing intermolecular interactions, mainly hydrogen bonds between the hydroxyl and carbonyl groups of APG and the ether and amide functionalities of SOL.

Rosiak et al. [37] studied an amorphous solid dispersion of apigenin (APG) with sodium alginate (SA), Pluronic^®^ F-68 (PLU68), Pluronic^®^ F-127 (PLU127), PVP K30, and PVP VA64 obtained by ball milling. **XRPD** analysis confirmed the complete alteration of APG’s crystalline structure to an amorphous form for dispersion with SA, PVP K30, and PVP VA64. In the case of dispersion with Pluronic^®^ after the amorphization process, the crystalline peaks of PLU68 and PLU127 were still observed, while the APG peaks disappeared. This indicates the complete dispersion of APG within the semicrystalline PLU matrix. The study reported in [125] confirmed that Pluronic^®^ is not entirely amorphous, with crystalline regions primarily composed of PEO layers, while the amorphous regions include both PPO and PEO. **SEM** analysis provided insights into the surface morphology of apigenin, Pluronic F-127, Pluronic F-68, and their dispersions, revealing distinct differences between the samples. The crystalline nature of APG was evident from its irregularly shaped particles with sharp edges, while the dispersions exhibited significant morphological changes. The smaller particle size in the APG-PLU127 dispersion contributed to an increased contact area with the solvent, enhancing the dissolution rate of APG compared to APG-PLU68. Additionally, the smooth surface of the dispersion influenced the release profile, leading to a slower release of APG. The thermal analysis confirmed the stability of APG and APG-PLU dispersions up to approximately 380 °C, with the melting point of APG observed at 366.3 °C. **DSC** measurements further characterized the thermal properties of the dispersions, revealing that the endothermic peak of APG was absent in the DSC curves of both physical mixtures and dispersions. The authors suggested the formation of a molecularly dispersed system, likely due to the dissolution of APG in the molten Pluronic^®^ during preparation. Additionally, the Tm of Pluronic^®^ shifted to lower temperatures in the dispersions (54.8 °C for APG-PLU68 BM and 56.2 °C for APG-PLU127 BM), while no such shift was observed in the physical mixtures. Based on this, the authors indicated the presence of chemical interactions between APG and Pluronic^®^ in the dispersions. **FT-IR** analysis confirmed the formation of hydrogen bonds between the phenolic groups of APG and the oxygen atoms of PLU.

Altamimi et al. [38] studied an amorphous solid dispersion of apigenin (APG) with Pluronic F-127 (1:1, 1:2, and 1:4 mass ratio) by spray-drying method. **XRPD** analysis confirmed the complete alteration of APG’s crystalline structure to an amorphous form only for a 1:4 mixture ( “halo” effect in the diffractogram). This was also confirmed by **DSC** analysis (disappearance of melting point for apigenin). **FT-IR** analysis indicated no change in the chemical structure of apigenin after the spray-drying process. In addition, it was indicated that hydrogen bonds were formed between the hydroxyl group of APG and the ether group of the polymer. **SEM** confirmed that particles in the obtained dispersions had leaf-like structures.

**Baicalein (BAC)**

Ding et al. [39] studied a co-amorphous solid dispersion (co-ASD) of BAC and nicotinamide (NIC) obtained by solvent evaporation. **XRPD** analysis confirmed the complete alteration of BAC’s crystalline structure to an amorphous form in BAC-NIC (“halo” effect in the diffractogram). **DSC** confirmed the formation of a single-phase co-ASD (glass transition event observed at 42.5 °C). **SEM** images of co-ASD revealed an irregular plate-like shape. **FT-IR** analysis suggested three intermolecular hydrogen bonds between BAC and NIC (–OH and N (in the pyridine ring of NIC), –OH and NH (in the amide group of NIC), and -O (in the hydroxyl group of BAC) and NH (in the pyridine ring of NIC)). **PLM** images of crystalline and amorphous forms of BAC differed in their visual appearance. The co-ASD form presented a plate-like shape with a smooth surface and no visible birefringence phenomenon, whereas strong birefringences were observed for plate-like crystalline BAI and dot-like crystalline NIC.

Jangid et al. [40] studied a co-amorphous solid dispersion (co-ASD) of BAC and amino acids (citric acid (CA), fumaric acid (FA), oxalic acid (OA), glutamic acid (GA), asparagina (ASP), and histidine (HIS)) obtained by solvent evaporation. **XRPD** analysis confirmed the complete alteration of BAC’s crystalline structure to an amorphous form in BAC-HIS (“halo” effect in the diffractogram). This was also confirmed by **DSC** analysis (shifting of BAC’s melting point and an observed glass transition temperature of 38 °C). **FT-IR** analysis confirmed intermolecular hydrogen bonds between BAC and HIS (peak corresponding to the –OH bond in BAC shifted and transformed into shoulder peaks). Moreover, a shift in the >C=O stretching frequencies of HIS suggested the involvement of this moiety in interaction with BAC. The results of stability studies confirmed that BAC-HIS co-ASD showed no indication of recrystallization within the 6-month study period. The high physical stability can be attributed to strong hydrogen bonding and π–π interactions between BAC and HIS.

Zhang et al. [41] studied an amorphous solid dispersion of baicalein (BAC) obtained by hot-melt extrusion. Based on a preliminary formulation study, the authors selected Kollidon VA 64 (PVP VA64) and Eudragit EPO (EPO) as carriers and Cremophor RH as the plasticizer. **XRPD** analysis confirmed the complete alteration of BAC’s crystalline structure to an amorphous form in the BAC-PVP VA64 system and BAC- EPO system (“halo” effect in the diffractogram). This was also confirmed by **DSC** analysis (disappearance of melting point for BAC). **FT-IR** analysis confirmed intermolecular hydrogen bonds between BAC and the carrier after extrusion (broadening peak at 3449.4 cm^−1^). Moreover, the changes observed in the range of 1500 cm^−1^ to 1600 cm^−1^ (broadening and lower intensity of peaks) indicated a stronger interaction between BAC and EPO than between BAC and PVP VA64. The results of stability studies confirmed that ASDs of BAC were sensitive to temperature and humidity (the content of ASDs remained uniform, but the dissolution decreased evidently). The authors confirmed that a suitable package to prevent moisture and a suitable temperature are very important for BAC solid dispersions.

**Butein (BUT)**

Kim et al. [42] studied a solid dispersion of BUT with polymer (PVP K30 and polaxamer 407) obtained by solvent evaporation. **XRPD** analysis confirmed the complete alteration of BUT’s crystalline structure to an amorphous form in BUT-PVP30 1:5 and BUT-PVP30-polaxamer 407 1:5:1 (disappearance of Bragg peaks characteristic of BUT). This was also confirmed by the **DSC** analysis (disappearance of melting point for BUT observed at 225.11 °C in pure compound).

**Chrysin**

Wang et al. [43] developed a stable ASD of chrysin with Plasdone^®^ S630 (hydrophilic carrier) by solvent evaporation method. **XRPD** analysis confirmed the complete alteration of chrysin’s crystalline structure to an amorphous form (disappearance of Bragg peaks characteristic of chrysin). Physical stability studies showed that the ASD was susceptible to high temperature and humidity.

Lee et al. [44] studied binary solid dispersions of chrysin with hydrophilic carriers (TPGS, Kolliphor^®^ HS 15, Brij^®^ L4, and poloxamer 407) and ternary solid dispersions with Brij^®^ L4 and aminoclay by solvent evaporation method. Based on a preliminary formulation study, the authors selected Brij ^®^L4 and aminoclay because the ternary solid dispersion was most effective in increasing the solubility of chrysin. **XRPD** analysis confirmed the complete alteration of chrysin’s crystalline structure to an amorphous form (disappearance of Bragg peaks characteristic of chrysin) in the chrysin + Brij^®^ L4 + aminoclay solid dispersion (1:3:5 weight ratio). This was also confirmed by **DSC** analysis (disappearance of melting point for chrysin observed at 288 °C in pure compound). In addition, **SEM** images indicated the amorphous state of chrysin in a solid dispersion. **SEM** confirmed a homogeneous blend of all ternary components in irregularly shaped particles.

**Chrysosplenol C (CRSP)**

Ng et al. [126] investigated the formulation of CRSP in a solid dispersion (SD) using hydrophilic carriers including PVP K-25 and PEG 6000 to enhance its solubility and dissolution properties. **XRPD** analysis confirmed the transformation of CRSP from its crystalline state into an amorphous form within the polymeric matrix, indicating successful dispersion. The **DSC** thermograms showed the disappearance of the characteristic endothermic melting peak of CRSP in the SD formulations, indicating its transformation into an amorphous state. Additionally, a decrease in the melting temperature was observed in physical mixtures P3 (188.64 °C), PM SP4 (185.68 °C), and SD SP4 (194.44 °C), suggesting possible interactions between CRSP and the polymeric carriers. **SEM** analysis revealed morphological changes, with CRSP appearing to be embedded in the polymeric matrix, which contributed to increased wettability and solubility. **FT-IR** spectroscopy suggested hydrogen-bonding interactions between the drug and polymeric carriers.

**Curcumin (CUR)**

Fan et al. [45] investigated the preparation of amorphous solid dispersions (ASDs) of CUR using hot-melt extrusion (HME) with EudragitE PO (EPO) as a carrier. The study aimed to enhance the solubility and bioavailability of the poorly water-soluble and thermosensitive polyphenol. To optimize the process, the authors examined the effects of barrel temperature, screw speed, and cooling methods on the physicochemical properties of the dispersions. **XRPD** analysis confirmed the complete transformation of CUR from its crystalline to amorphous form within the EPO matrix. **DSC** further demonstrated the disappearance of the characteristic endothermic peak of CUR, indicating molecular dispersion within the polymer. **FT-IR** suggested the presence of hydrogen-bond interactions between the phenolic groups of CUR and the polymer matrix, contributing to stabilization in the amorphous state.

Fan et al. [46] investigated the formulation of curcumin (CUR) amorphous solid dispersions (ASDs) using binary polymer systems—specifically, Eudragit EPO combined with either polyvinylpyrrolidone K30 (PVP) or hydroxypropyl methylcellulose E50 (HPMC). The study focused on elucidating the molecular interactions and wetting properties of these dispersions to determine their influence on CUR dissolution and stability. **FT-IR** and **Raman imaging spectroscopy** confirmed hydrogen-bond formation between CUR and the polymers, with stronger interactions observed in the CUR/EuD-HPMC system compared to CUR/EuD-PVP. **Molecular docking** studies further supported these findings, indicating that HPMC formed a higher number of hydrogen bonds with CUR, particularly with its carbonyl and hydroxyl groups. **XRPD** analysis demonstrated the successful amorphization of CUR in both formulations, with no crystalline peaks detected in the ASDs.

Mai et al. [47] explored the development of ASDs of CUR using vibrational ball milling with hydroxypropyl cellulose (HPC) and/or sodium dodecyl sulfate (SDS) as carriers. The study aimed to enhance the solubility and stability of CUR. **XRPD** confirmed the amorphization of CUR in dispersions containing 90% HPC, while formulations with SDS facilitated a faster transformation to the amorphous state. **DSC** demonstrated the disappearance of the CUR melting peak, indicating molecular dispersion within the polymer matrix. **FT-IR** revealed the formation of hydrogen bonds between CUR and HPC, contributing to the stabilization of the amorphous structure. **Stability studies** indicated that the amorphous state remained stable for up to 30 days at 40 °C and 75% relative humidity, with partial recrystallization occurring after 60 days.

Fan et al. [48] investigated the preparation of a CUR sustained-release solid dispersion (CUR-SD) using HME with Eudragit RSPO and Eudragit RLPO as polymeric carriers. The study aimed to optimize the formulation and processing parameters, including barrel temperature, screw speed, and cooling rate, to enhance curcumin’s solubility and bioavailability. The amorphous nature of curcumin in the solid dispersion was confirmed through DSC and PXRD analyses. **DSC** thermograms revealed the disappearance of the sharp endothermic peak characteristic of crystalline curcumin (observed at 185.4 °C), indicating the transition to an amorphous state within the polymer matrix. Similarly, **XRPD** patterns demonstrated the absence of distinct diffraction peaks in the solid dispersions, showing a broad halo pattern instead, which is typical for amorphous materials. These findings confirm that curcumin was molecularly dispersed in the Eudragit carriers. **Stability tests** over six months showed no significant changes in the solid dispersion. In vitro dissolution studies demonstrated a sustained-release profile controlled by diffusion and dissolution mechanisms.

Huang et al. [49] investigated the preparation of an amorphous surfactant-free solid dispersion (ASD) of CUR using chitosan oligosaccharide (COS) as the amorphous matrix. The study aimed to enhance the solubility and permeability of CUR by ball milling with COS at different weight ratios (1:1, 1:2, and 1:4). The amorphous nature of CUR in the ASD was confirmed through DSC and XRPD analyses. **DSC** thermograms showed the disappearance of the sharp endothermic peak of crystalline CUR (181.6 °C) in the ASD samples, indicating a transition to an amorphous form. **XRPD** patterns further supported this finding, as the characteristic crystalline diffraction peaks of CUR were absent in the ASD, replaced by a broad halo pattern typical of amorphous materials.

He et al. [50] investigated the molecular interactions in curcumin–polymer complexes to enhance the anti-inflammatory effects of curcumin. Amorphous solid dispersions (ASDs) of curcumin were prepared using different polymers, including polyvinylpyrrolidone (PVP), poloxamers, and hydroxypropyl-β-cyclodextrin (HP-β-CD), via solvent evaporation. The study demonstrated that PVP-based ASDs exhibited superior solubility and stability compared to poloxamer- and HP-β-CD-based formulations due to strong drug–polymer interactions. Comprehensive characterization using XRD, DSC, FT-IR, SEM, Raman, and ^1^H-NMR confirmed the molecule-level dispersion of curcumin and the presence of intermolecular hydrogen bonding. **XRPD** and **DSC** analyses confirmed the transformation of crystalline curcumin into an amorphous state in polymer-based solid dispersions. **FT-IR** and **Raman spectroscopy** identified hydrogen bonding between curcumin and polymers, with shifts in characteristic peaks indicating molecular interactions. **SEM** revealed significant morphological changes, including reduced particle size and smoother surfaces, contributing to enhanced dissolution. **^1^H-NMR** confirmed molecule-level dispersion, showing shifts in proton signals due to hydrogen bonding and inclusion-complex formation, particularly with HP-β-CD. **Molecular dynamics simulations** further elucidated the binding mechanisms, highlighting the role of hydrophobic interactions and hydrogen bonding in enhancing curcumin’s stability and bioavailability.

Shin et al. [51] developed a hydroxypropyl methylcellulose (HPMC)-based amorphous solid dispersion of CUR (DW-CUR 20) to enhance its bioavailability and hepatoprotective activity. **DSC** and **XRPD** analyses confirmed the amorphization of curcumin in the dispersion, while **SEM** showed morphological changes contributing to improved solubility.

Fan et al. [52] investigated the role of hydroxypropyl methylcellulose (HPMC) in maintaining the stability and enhancing the solubilization of curcumin amorphous solid dispersions (Cur ASDs) formulated with Eudragit E100 (E100). **XRPD** analysis confirmed the successful amorphization of curcumin, as the characteristic crystalline peaks observed in pure curcumin disappeared entirely in Cur ASDs, indicating a homogeneous dispersion of curcumin within the polymer matrix. This was further supported by **DSC** analysis, where the sharp endothermic peak of pure curcumin at ~180 °C, corresponding to its melting point, was absent or significantly reduced in the dispersions, confirming the loss of crystallinity and suggesting the formation of a molecularly dispersed system. Additionally, the shift in the polymer’s glass transition temperature (Tg) in the dispersions indicated interactions between curcumin and the polymer matrix, contributing to enhanced physical stability. A **stability study** revealed that HPMC effectively inhibited recrystallization, as confirmed by extended dissolution studies after six months.

Fan et al. [127] investigated the impact of hydroxypropyl methylcellulose (HPMC) on the inhibition of crystallization and improvement of permeability in CUR ASDs formulated with Eudragit E100 (E100). **XRPD** analysis confirmed the successful amorphization of CUR, as the characteristic crystalline peaks observed in pure CUR disappeared entirely in Cur ASDs, indicating a homogeneous dispersion within the polymer matrix. **DSC** analysis further supported this, showing the absence of or significant reductions in curcumin’s sharp endothermic melting peak at ~180 °C in the dispersions, confirming the loss of crystallinity and the formation of a molecularly dispersed system. Additionally, the shift in the polymer’s glass transition temperature (Tg) suggested interactions between curcumin and the polymer matrix, contributing to improved physical stability.

Li et al. [53] investigated the molecular and interfacial interactions in curcumin amorphous solid dispersions (Cur ASDs) formulated with various polymers, including polyethylene glycol (PEG), polyvinylpyrrolidone (PVP), Eudragit EPO (EuD), EuD/hydroxypropyl methylcellulose (HPMC), and PVP/EuD, to better understand the dissolution mechanisms. **XRPD** analysis confirmed the successful amorphization of curcumin, as the characteristic crystalline peaks observed in pure curcumin disappeared in the dispersions, indicating a homogeneous distribution within the polymer matrices. **DSC** analysis further supported these findings by showing the absence of or significant reductions in curcumin’s sharp endothermic melting peak at ~180 °C, confirming the loss of crystallinity and the formation of a molecularly dispersed system. Additionally, shifts in the glass transition temperature (Tg) of the polymer matrices suggested intermolecular interactions between curcumin and the excipients, which contributed to improved physical stability.

Kadota et al. [54] developed a hydrolysis-resistant solid dispersion system of CUR using α-glucosyl hesperidin (hesperidin-G) and polyvinylpyrrolidone (PVP K-30) via the solvent evaporation method. The study aimed to enhance CUR solubility while preventing its rapid hydrolytic degradation in aqueous environments. DSC and XRPD analyses confirmed the amorphous nature of CUR in the ternary system. **DSC** thermograms showed the disappearance of the characteristic endothermic peak of crystalline CUR at 188 °C, indicating its conversion to an amorphous form. Similarly, **XRPD** analysis revealed the absence of distinct diffraction peaks corresponding to crystalline CUR, replaced by a broad halo pattern typical of amorphous materials. These results suggest that CUR was molecularly dispersed within the hesperidin-G/PVP K-30 matrix.

Kadota et al. [55] developed a tri-component solid dispersion system of curcumin (CUR) with α-glucosyl stevia (Stevia-G) and polyvinylpyrrolidone (PVP) using freeze-drying to enhance the solubility, oral absorption, and photochemical stability of CUR. The study aimed to improve the poor bioavailability and rapid degradation of CUR by incorporating these excipients to stabilize its amorphous state. DSC and XRPD analyses confirmed the amorphous nature of CUR in the ASD. **DSC** thermograms showed the disappearance of the sharp endothermic peak corresponding to crystalline CUR’s melting point (approximately 180 °C), indicating its transition to an amorphous state. **XRPD** patterns further supported this, as the characteristic crystalline diffraction peaks of CUR were absent in the tri-component system, replaced by a broad halo pattern typical of amorphous materials.

Kerdsakundee et al. [56] developed a gastroretentive raft-forming system incorporating curcumin–Eudragit EPO solid dispersions (CUR-EPO SD) to enhance curcumin’s solubility and provide sustained drug release for gastric ulcer treatment. Solid dispersions of CUR with EudragitEPO were prepared using the solvent evaporation method at different weight ratios, with a 1:5 ratio identified as optimal for incorporation into the raft-forming system. DSC and XRPD analyses confirmed the amorphous nature of CUR in the dispersions. **DSC** thermograms showed the disappearance of the sharp endothermic peak corresponding to crystalline CUR (observed at ~188 °C), indicating a transition to an amorphous form. **XRPD** analysis further supported this finding, as the characteristic crystalline diffraction peaks of CUR were absent in the CUR-EPO SDs, replaced by a broad halo pattern indicative of amorphization.

Li et al. [57] developed a curcumin–Eudragit EPO solid dispersion (Cur@EPO) using a simple solution-mixing method to address the low solubility, poor stability, and limited bioavailability of curcumin. The study aimed to enhance curcumin’s aqueous solubility, protect it from degradation, and evaluate its potential for transdermal delivery. DSC and PXRD analyses confirmed the amorphous nature of curcumin in the solid dispersion. **DSC** thermograms showed the disappearance of crystalline curcumin’s characteristic melting peak (178.8 °C), indicating its transition to an amorphous state. **XRPD** patterns further supported this finding, as the sharp crystalline peaks of curcumin were absent in Cur@EPO, replaced by a broad halo pattern characteristic of amorphous materials. **Stability tests** demonstrated that Cur@EPO provided significant protection against hydrolysis at alkaline pH and degradation under UV exposure, with more than 85% of curcumin preserved after 24 h of UV treatment. **FT-IR** and **^1^H NMR** confirmed hydrogen-bonding interactions between the hydroxyl groups of curcumin and the carbonyl groups of EudragitEPO.

Gangurde et al. [58] developed curcumin–Eudragit EPO solid dispersions (CUR-EuD SDs) using spray-drying and rotary evaporation techniques to enhance the solubility and dissolution of curcumin. The study also included in silico molecular modeling to elucidate drug–polymer interactions at the molecular level. DSC and XRPD analyses confirmed the amorphous nature of curcumin in the solid dispersion. **DSC** thermograms showed the disappearance of the sharp endothermic peak of crystalline curcumin (~179.92 °C), indicating its transition to an amorphous state. **XRPD** analysis further supported this finding, as the characteristic crystalline peaks of curcumin were absent in the SD formulations, replaced by a broad halo pattern indicative of amorphization. Further **molecular docking** and **molecular dynamics simulations** provided insights into the binding interactions between curcumin and Eudragit EPO. The study highlighted the importance of van der Waals interactions in drug–polymer binding, while hydrogen bonding played a lesser role. The findings confirmed that Eudragit EPO effectively stabilized the amorphous form of CUR and prevented recrystallization.

Chuah et al. [59] developed an amorphous solid dispersion (ASD) of curcumin (CUR) using hot-melt extrusion with hydroxypropyl methylcellulose (HPMC), lecithin, and isomalt to enhance its solubility, bioavailability, and bio-efficacy for functional food applications. The formulation aimed to address the poor oral bioavailability of curcumin due to its low solubility and rapid metabolism. DSC and PXRD analyses confirmed the amorphous nature of curcumin in the ASD. **DSC** thermograms showed the disappearance of the sharp endothermic peak characteristic of crystalline CUR (~172 °C), indicating its transition to an amorphous state. **XRPD** analysis further supported this finding, as the characteristic crystalline peaks of CUR were absent in the ASD formulation, replaced by a broad halo pattern indicative of amorphization.

Wegiel et al. [60] investigated the physical stability of amorphous curcumin solid dispersions (CUR-ASDs) and the role of intra- and intermolecular hydrogen bonding in preventing crystallization. The study explored the ability of various polymers—including polyvinylpyrrolidone (PVP), EudragitE100, carboxymethyl cellulose acetate butyrate (CMCAB), hydroxypropyl methylcellulose (HPMC), and HPMC-acetate succinate (HPMCAS)—to stabilize amorphous curcumin. DSC and PXRD analyses confirmed the amorphous nature of curcumin in the solid dispersions. **DSC** thermograms showed the disappearance of the sharp melting peak of crystalline curcumin (~183 °C), indicating its transition to an amorphous state. **XRPD** patterns further supported this finding, as the characteristic crystalline peaks of curcumin were absent in the dispersions, replaced by a broad halo pattern typical of amorphous materials. **Stability studies** demonstrated that the polymers effectively inhibited curcumin crystallization to varying degrees, with EudragitE100 providing the highest stability due to ionic interactions between its basic groups and the phenolic groups of curcumin. **FT-IR** analysis confirmed the presence of hydrogen-bonding interactions between curcumin and polymers, although curcumin’s intrinsic intramolecular hydrogen bonding limited the extent of these interactions. **Storage studies** under different temperature and humidity conditions revealed that amorphous curcumin was highly prone to recrystallization without polymeric stabilization. PVP was the least effective crystallization inhibitor, while EudragitE100 demonstrated the longest inhibition period.

Li et al. [61] developed ASDs of CUR using cellulose-derived polymer matrices, including hydroxypropyl methylcellulose acetate succinate (HPMCAS), carboxymethyl cellulose acetate butyrate (CMCAB), and cellulose acetate adipate propionate (CAAdP), with the aim of enhancing CUR’s aqueous solubility and chemical stability. The study focused on elucidating structure–property relationships between polymer characteristics and their ability to inhibit CUR’s crystallization, enhance solution concentration, and stabilize CUR against degradation in aqueous environments. DSC and XRPD confirmed the amorphous nature of CUR within all solid dispersions. **DSC** thermograms showed the disappearance of the sharp melting endotherm of crystalline CUR (≈177–183 °C), indicating successful molecular dispersion of CUR in the polymer matrices. **XRPD** patterns further corroborated these findings, as the characteristic diffraction peaks of crystalline CUR were absent and replaced by a broad halo typical of amorphous materials. Even at high CUR loadings (up to a 9:1 CUR/polymer ratio), the dispersions remained amorphous (confirmed by XRPD), demonstrating strong inhibition of crystallization by the cellulose-based polymers. **FT-IR** provided evidence of specific drug–polymer interactions responsible for amorphous stabilization. FT-IR spectra of CUR–polymer dispersions showed significant broadening and shifts of the hydroxyl stretching bands of CUR (~3500–3300 cm^−1^) compared with crystalline and amorphous CUR alone, indicating changes in hydrogen-bonding environments. In addition, shifts of polymer carbonyl stretching bands to lower wavenumbers (e.g., from ~1743 to ~1737 cm^−1^ for HPMCAS-based dispersions) were observed, suggesting the formation of hydrogen bonds between CUR hydroxyl groups and polymer carbonyl groups. These spectral changes could not be reproduced by simple superposition of individual component spectra, confirming the presence of specific intermolecular interactions rather than mere physical mixing.

Onoue et al. [62] developed a nanocrystal solid dispersion (CSD-Cur) of CUR. The study also compared CSD-Cur with amorphous solid dispersion (ASD-Cur) and nanoemulsion (NE-Cur) formulations to assess their physicochemical and pharmacokinetic properties. DSC and PXRD analyses confirmed the crystallinity of CUR in the CSD formulation and the amorphous nature of CUR in the ASD. **DSC** thermograms showed the characteristic melting peak of crystalline CUR at ~180 °C, which was retained in CSD-Cur but absent in ASD-Cur, indicating its amorphization. **XRPD** analysis further supported these findings, as CSD-Cur displayed sharp crystalline peaks, whereas ASD-Cur exhibited a broad halo pattern, confirming its amorphous state. **Photostability tests** indicated that CUR in solution was highly photoreactive and degraded rapidly under UV exposure. However, solid-state formulations—particularly CSD-Cur—provided superior photostability, with only 17% degradation after intense UV irradiation. In contrast, ASD-Cur degraded by ~50%, and NE-Cur exhibited significant photodegradation.

Paradkar et al. [63] developed curcumin–polyvinylpyrrolidone (CUR-PVP) solid dispersions (SDs) using spray drying. The study aimed to overcome the poor aqueous solubility and rapid degradation of curcumin at alkaline pH by formulating it in different CUR:PVP ratios (1:1, 1:3, 1:5, 1:7, and 1:10). DSC and PXRD analyses confirmed the amorphous nature of CUR in the SDs. **DSC** thermograms showed the disappearance of the characteristic melting peak of crystalline curcumin (~180 °C) in the SD formulations, indicating a transition to an amorphous state. **XRPD** patterns further supported this, as the sharp diffraction peaks of crystalline curcumin disappeared in the SDs, replaced by a broad halo pattern characteristic of amorphous materials.

**Curcumin–Hesperetin (CUR-HSP)**

Wdowiak et al. [64] developed amorphous solid dispersions (ASDs) of CUR and HSP using polyvinylpyrrolidone K30 (PVP K30) and phosphatidylcholine as carriers, prepared via hot-melt extrusion (HME). Formulation parameters were optimized using a Box–Behnken experimental design. DSC and XRPD analyses confirmed the amorphous nature of CUR and HSP in the dispersions. **DSC** thermograms showed the disappearance of the melting peaks of crystalline CUR (~183 °C) and HSP (~232 °C), indicating their transition to an amorphous state. **XRPD** patterns further supported these findings, as the sharp crystalline peaks of CUR and HSP were absent in the ASD formulations, replaced by a broad halo pattern typical of amorphous materials. **FT-IR** analysis suggested intermolecular interactions between the active compounds and the carriers, contributing to solubility enhancement and physical stability.

**Curcumin–Magnolol**

Han et al. [65] prepared a co-amorphous CUR–magnolol (CUR-MAG) system with small amount of polymer–hydroxypropyl methylcellulose (HPMC), hydroxypropyl cellulose (HPC), or polyvinylpyrrolidone K30 (PVP K30) by quench cooling —incorporated at 5% (*w*/*w*) to form ternary co-amorphous systems. DSC and XRPD analyses confirmed the amorphous nature of CUR-MAG. **DSC** thermograms showed the disappearance of the characteristic melting peaks of crystalline CUR (~183 °C) and MAG (~101 °C), indicating the formation of a single-phase amorphous system. **XRPD** patterns further supported this finding, as the sharp crystalline peaks of CUR and MAG were absent in the co-amorphous formulations, replaced by a broad halo pattern characteristic of amorphous materials. **Storage stability studies** showed that the ternary co-amorphous systems exhibited enhanced physical stability, with delayed recrystallization under accelerated conditions (40 °C/75% RH). **FT-IR** and **ss-NMR** analyses suggested the involvement of molecular interactions between the polymers and CUR-MAG, which contributed to improved stability.

**Curcumin-Piperine (CUR-PIP)**

Wdowiak et al. [31] developed ternary amorphous systems of CUR and **PIP** using hme with Kollidon VA64 (vinylpyrrolidone-vinyl acetate copolymer) as a polymeric carrier and crystallization inhibitor. Kollidon VA64 was selected due to its proven ability to inhibit recrystallization, improve wettability, and stabilize supersaturated solutions. The amorphous nature of the extruded systems was confirmed by DSC and XRPD analyses. **DSC** thermograms of the amorphous dispersions showed the disappearance of the characteristic melting endotherms of crystalline CUR (≈184 °C) and PIP (≈133 °C), while a single glass transition temperature (Tg) was observed for each formulation, indicating molecule-level mixing of all components. **XRPD** patterns further supported complete amorphization, as sharp crystalline diffraction peaks of CUR and PIP were absent and replaced by broad halo patterns typical of amorphous materials. Increasing polymer content led to higher Tg values, suggesting reduced molecular mobility and improved physical stability of the systems. FT-IR was used to investigate intermolecular interactions between CUR, PIP, and Kollidon VA64. Significant band broadening, shifts, and intensity changes were observed in the spectra of amorphous systems compared with crystalline and amorphous raw compounds. In particular, shifts in characteristic CUR bands (e.g., around 1577–1586 cm^−1^ and 1119–1123 cm^−1^) and PIP bands (≈1502–1485 cm^−1^) suggested the formation of specific intermolecular interactions, most likely hydrogen bonding and dipole–dipole interactions between the active compounds and the polymer carrier. These interactions were identified as key factors contributing to the physical stability of the amorphous state and inhibition of recrystallization during storage.

**Daidzein**

Panizzon et al. [66] studied different types of solid dispersions of daidzein (DAI) (second-generation solid dispersions (SG), third-generation solid dispersions (TG), and second- and third-generation pH-modulated solid dispersions) prepared by spray drying. **XRPD** analysis confirmed the complete alteration of DAI’s crystalline structure to an amorphous form (disappearance of Bragg peaks characteristic of daidzein) in all solid dispersions. This was also confirmed by the **DSC** analysis (disappearance of melting point for DAI observed at 336 °C in pure compound). In addition, **SEM** imaging indicated the amorphous state of DAI in solid dispersion (solid dispersion: smooth surface and concave depressions (collapsed walls), with no agglomerates or pinholes; DAI crystals: prismatic shape with similar sizes).

**Diosmin**

Anwer et al. [67] studied ASDs of diosmin (DSM) with Soluplus (SOL) in different weight ratios (1:0.5, 1:1 and 1:2 *w*/*w*) prepared by spray drying. **XRPD** diffractograms of all spray-dried solid dispersions showed some characteristic peaks of DSM. These results indicate a partially amorphous form of DSM. The relative degrees of crystallinity of spray-dried powders of DSM-SOL were 0.366 (1:0.5 *w*/*w*), 0.233 (1:1 *w*/*w*), and 0.161(1:2 *w*/*w*). Increasing the amount of SOL in the solid dispersion increased the amorphous character of the powder. This was also confirmed by **DSC** analysis. DSM exhibited a sharp endothermic effect at 273 °C, corresponding to the melting point. Complete disappearance of the melting point was not observed in any of the solid dispersions. **SEM** imaging was performed only for the 1:2 *w*/*w* solid dispersion. This dispersion had a spherically shaped particle with a wrinkle and a contracted surface due to loss of moisture. In addition, SEM confirmed the aggregation of particles (probably due to SOL). **FT-IR** spectra of all dispersions showed a decrease in the intensity of peaks in the fingerprint region (400–1600 cm^−1^), confirming the possible entrapment of DSM inside the SOL matrix.

**Ellagic acid**

Li et al. [68] studied ASDs of ellagic acid (EA) with different polymers prepared by spray-drying (EA with CMCAB, HPMCAS, and PVP), co-precipitation (EA with CAAdP), and solvent evaporation (EA with CAAdP and PVP) methods in 1:3 and 1:9 weight ratios. **XRPD** analysis confirmed the complete alteration of ellagic acid’s crystalline structure to an amorphous form in all solid dispersions, except for EA/CMCAB and EA/CAAdP 1:3 *w*/*w* solid dispersions (effect “halo” in diffractograms). **Modulated DSC** determined Tg values based on reversible heat flows. The publication fully characterized only **FT-IR** spectra of EA/HPMCAS solid dispersions. Results showed that a small, sharp peak at 3558 cm^−1^ observed in EA spectra corresponding to the OH stretching vibration disappeared in the spectra of solid dispersions. Next, changes related to C=O stretching vibrations were observed in EA at 1699 cm^−1^ (peak disappeared) and HPMCAS at 1739 cm^−1^ (peak shifted slightly to 1741 cm^−1^). These changes indicate that the carbonyl groups of both HPMCAS and EA are involved in H-bonding interactions.

**Epigallocatechin gallate**

Cao et al. [69] studied ASDs of epigallocatechin gallate (EGCG) obtained by freeze-drying method. Based on a preliminary formulation study, the authors selected HPMCAS, HPMCP, Soluplus^®^, and cellulose acetate as carriers. **XRPD** analysis confirmed the complete alteration of EGCG’s crystalline structure to an amorphous form (disappearance of Bragg peaks characteristic of EGCG) in all solid dispersions. Lyophilized pure EGCG was also amorphous (“halo” effect in the diffractogram). **SEM** imaging indicated disparate morphologies in all samples. The crystalline form of EGCG was characterized by long, thin, flat laths, while the amorphous lyophilized EGCG had a continuous structure consisting of round-ended fibers and small spheres, whereas amorphous dispersions had continuous structures with different morphologies. **mDSC** analysis confirmed the miscibility of EGCG-HPMCAS and EGCG-Soluplus^®^ dispersions (one apparent T_g_) and suggested that EGCG-HPMCP and EGCG–cellulose acetate dispersions are not miscible (irregular results). These results were consistent with the observations of microstructural morphologies obtained by SEM. **Physical stability** studies (40 °C/75% RH, 11 days) confirmed that amorphous lyophilized EGCG was not stable under stressed conditions (observed recrystallization), whereas four amorphous dispersions were physical stable under the same stressed condition. Visual observation after 11 days indicated that EGCG-Soluplus^®^ was chemically stable (retaining its white color), unlike the other three dispersions (dispersions presented different levels of color changes). The authors indicated that the color change of EGCG is directly related to its chemical instability, i.e., degradation and oxidation. **TG** analysis confirmed certain amounts of volatiles in all samples. On the basis of these results, the authors estimated the amount of volatiles and accounted for them when calculating the EGCG equivalency in dissolution testing.

**Ferulic acid (FA)**

Albuquerque [70] studied ASDs of ferulic acid (FA) with different polymers (HPMC, Soluplus, PVP VA64, PVP K30, and PEG 6000) prepared by kneading, physical mixture, and solid evaporation. The prepared systems were characterized by **XRPD**, **FT-IR**, **SEM**, and **DSC**. Ultimately, complete amorphization was not obtained. The most promising results were shown by the Soluplus system, in which a diffractogram confirmed the significant disappearance of the crystalline bands characteristic of ferulic acid.

Huang et al. [71] developed ternary amorphous solid dispersions (SDs) of ferulic acid (FA) using coaxial electrospinning to enhance its solubility and dissolution rate. The study compared these third-generation SDs (3rd SDs) with second-generation SDs (2nd SDs) prepared using traditional single-fluid blending electrospinning to assess the impacts of polymer selection and processing technique on FA performance. DSC and XRPD analyses confirmed the amorphous nature of FA in both SD types. **DSC** thermograms showed the disappearance of the sharp melting peak of crystalline FA (~174 °C), indicating its transition to an amorphous state. **XRPD** patterns further supported this, as the characteristic crystalline peaks of FA were absent in the SDs, replaced by a broad halo pattern typical of amorphous materials.

Nadal et al. [72] studied solid dispersions of FA with different polymers (PVP, PEG 6000, and polaxamer-188) obtained by spray-drying method. XRPD analysis confirmed incomplete amorphization of the FA-PVP dispersion.

**Fisetin (FIS)**

Rosiak et al. [73] developed binary amorphous solid dispersions (ASDs) and ternary amorphous solid inclusions (ASIs) of fisetin (FIS) using a mechanochemical method to enhance its solubility and biological activity. The formulations incorporated EudragitL100 (EL100), EudragitEPO (EPO), and 2-hydroxypropyl-β-cyclodextrin (HPβCD) as carriers to improve the dissolution and stability of FIS. DSC and XRPD analyses confirmed the amorphous nature of FIS in the ASDs and ASIs. **XRPD** patterns showed the absence of characteristic diffraction peaks of crystalline FIS, replaced by a broad halo pattern typical of amorphous materials. **DSC** thermograms demonstrated the full miscibility of FIS within the polymeric matrices, indicating successful amorphization.

Skiba et al. [75] prepared ASDs of FIS with polymers based on cyclodextrins (poly-aβ-CD, poly-aγ-CD, poly-aβγ-CD, and poly-methyl-β-CD) by spray drying. **DSC** confirmed the formation of a miscible amorphous phase in the FIS-poly-methyl-β-CD ASD (the melting point of FIS disappeared completely). The **SEM** images revealed that FIS was uniformly incorporated into the poly-methyl-β-CD matrix, leading to the loss of its crystalline structure. An **NMR** study confirmed that the ASDs provide better solubilization of FIS compared to their corresponding physical mixtures. **FT-IR** analysis confirmed the formation of the inclusion complex (masking of the absorption peaks of FIS by those of poly-methyl-β-CD). The absence of new absorbance bands suggested that FIS interacted with poly-methyl-β-CD via noncovalent and weak intermolecular forces such as van der Waals forces or hydrogen bonds.

Sip et al. [74] developed an amorphous solid dispersion (ASD) of fisetin (FIS) using supercritical carbon dioxide (scCO_2_) with copovidone (CPV) as a co-former to enhance its solubility, dissolution rate, and biological activity. The study aimed to improve FIS’s bioavailability while investigating its antioxidant, neuroprotective, and microbiome-modulating properties. DSC and XRPD analyses confirmed the amorphous nature of FIS in the dispersion. XRPD patterns showed the disappearance of the characteristic crystalline peaks of FIS, replaced by a broad halo pattern, indicating successful amorphization. **FT-IR** spectroscopy further suggested intermolecular interactions between FIS and CPV, stabilizing the amorphous form. In particular, **FT-IR** analysis suggested that the C-O, C-OH, and/or –OH groups of FIS may form hydrogen bonds with the C=O and/or –OH groups of CPV, contributing to enhanced solubility and physical stability. A 3-month stability analysis at a temperature of 25 °C and 70% relative humidity (reflective of typical storage conditions) showed that the ASD was physically stable.

**Genistein (GEN)**

Novo et al. [76] investigated cellulose-based amorphous solid dispersions (ASDs) of GEN. The dispersions were prepared using a solvent-based method involving rotary evaporation, in which GEN and the respective cellulose derivatives were included: cellulose acetate glutarate (CAG), 5-carboxypentyl hydroxypropyl cellulose (CP-HPC), and hydroxypropyl methylcellulose acetate succinate (HPMCAS). These carriers were selected to systematically vary amphiphilicity and carboxylic acid content in order to elucidate structure–property relationships governing GEN solubilization and crystallization inhibition. The amorphous nature of GEN in the prepared dispersions was confirmed using DSC and XRPD. **DSC** thermograms showed the disappearance of the characteristic melting endotherm of crystalline GEN, while **XRPD** patterns revealed the absence of sharp diffraction peaks and the presence of broad amorphous halos, indicating successful molecular dispersion of GEN within the polymer matrices. FT-IR provided evidence of specific polymer–drug interactions, as band broadening and shifts in the hydroxyl stretching region of GEN were observed in the dispersions compared with crystalline GEN, consistent with hydrogen bonding between GEN phenolic groups and functional groups of the cellulose derivatives.

Garbiec et al. [34] developed co-amorphous systems of genistein (GEN) with basic amino acids—lysine (LYS) and arginine (ARG)—using mechanochemical activation to enhance GEN solubility, dissolution, and biological activity. The study aimed to assess the impact of these amino acids as co-formers on the physicochemical properties, stability, and bioactivity of GEN. DSC and PXRD analyses confirmed the amorphous nature of GEN in the co-amorphous systems. **XRPD** patterns showed the absence of characteristic crystalline diffraction peaks of GEN, replaced by a broad halo pattern, confirming successful amorphization. **DSC** thermograms demonstrated the disappearance of the sharp melting peak of crystalline GEN (~298 °C), indicating a transition to an amorphous state. **Stability studies** under accelerated conditions (40 °C/75% RH, 6-month storage duration) confirmed that GEN remained in the amorphous state, with no evidence of recrystallization over the tested period. FT-IR and Raman spectroscopy analyses suggested strong molecular interactions between GEN and LYS/ARG, stabilizing the amorphous state and preventing phase separation.

Zaini et al. [77] studied an ASD of GEN with PVP K30 prepared by solvent co-evaporation. **XRPD** analysis confirmed the complete alteration of GEN’s crystalline structure to an amorphous form for GEN-PVP K-30 (1:2 ratio) (“halo” effect in the diffractogram). This was also confirmed by **DSC** analysis (disappearance of the melting point for GEN observed at 303.4 °C in pure compound). **FT-IR** analysis confirmed intermolecular hydrogen bonds between GEN and PVP K30 (peak changes in the range of 3100–3400 cm^−1^ (OH groups in GEN)).

**Hesperetin (HES)**

Wdowiak et al. [78] studied amorphous inclusion complexes of HES with HP-β-CD prepared by solvent evaporation. **XRPD** analysis confirmed the complete alteration of HES’s crystalline structure to an amorphous form for HES/HP-β-CD in 1:1 and 1:2 molar ratios (halo effect in the diffractogram). This was also confirmed by **DSC** analysis (disappearance of melting point for HES observed at 234 °C in pure compound). **FT-IR** analysis confirmed the formation of hydrogen bonds between HES and HP-β-CD.

**Hesperetin–Naringenin (HES-NAR)**

Kanaze et al. [128] prepared nanodispersion-based solid dispersions of HES and NAR using PVP via the solvent evaporation method. **XRPD** analysis confirmed the loss of the long-range crystalline order of both flavanone aglycones in the PVP matrix, indicating their amorphous state. **DSC** analysis showed the disappearance of the characteristic melting endotherms of crystalline HES and NAR in the PVP nanodispersions. **SEM** analysis revealed homogeneous nanodispersed systems without detectable crystalline particles. **Stability studies** performed under accelerated conditions (40 °C/75% RH) confirmed that the PVP nanodispersions remained physically stable, showing no recrystallization and no changes in dissolution profiles during storage.

**Hesperetin–Piperine (HES-PIP)**

Wdowiak et al. [79] studied amorphous systems of HES-PIP with PVP VA64 prepared by ball milling. **XRPD** analysis confirmed the complete alteration of HES and PIP’s crystalline structures to an amorphous form for all obtained systems (“halo” effect in diffractograms). This was also confirmed by **DSC** analysis (disappearance of melting points for HES and PIP observed at 234.1 °C and 133.0 °C, respectively). **FT-IR analysis** could not clearly confirm the formation of hydrogen bonds. Amorphization improved the apparent solubility of HES and PIP by 245-fold and 183-fold, respectively. In addition, amorphization enhanced the dissolution rates of HES and PIP.

**Hesperidin (HED)**

Rosiak et al. [19] studied ASDs of HED with alginate sodium, HPMC, and Soluplus prepared by ball milling. **XRPD** analysis confirmed the complete alteration of HED’s crystalline structure to an amorphous form for HED-carrier 1:5 *w*/*w* (“halo” effect in the diffractogram). This was also confirmed by **DSC** analysis (one observed glass transition).

Wdowiak et al. [78] studied amorphous inclusion complexes of HED with HP-β-CD prepared by solvent evaporation. **XRPD** analysis confirmed the complete alteration of HED’s crystalline structure to an amorphous form for HED/HP-β-CD in 1:1 and 1:2 molar ratios (“halo” effect in diffractograms). This was also confirmed by **DSC** analysis (disappearance of melting point for HED observed at 259 °C in pure compound). **FT-IR** analysis confirmed the formation of hydrogen bonds between HED and HP-β-CD.

**Kaempferol (KMP)**

Rosiak et al. [32] studied ASDs of KMP with Eudragits (EPO, L100, L100-55) prepared by ball milling. **XRPD** analysis confirmed the complete alteration of KMP’s crystalline structure to an amorphous form for KMP-EL100 (20–50% KMP content), KMP-EL100-55 (20–30% KMP content), and KMP-EPO (20–60%) (“halo” effect in diffractogram). **DSC** analysis verified the complete miscibility of ASDs (a single glass transition temperature). **FT-IR** examination affirmed the establishment of hydrogen bonds between the –OH and/or –CH group of KMP and the C=O group within Eudragits.

**Luteolin (LUT)**

Koromili et al. [80] studied ASDs of LUT with different polymers (PVP K90, PVPVA64, Soluplus, HPC-SL, HPMCAS, and Eudragit EPO) prepared by film casting. **PLM** images confirmed that LUT-PVP K90 and LUT-PVP VA64 films have been recognized as promising matrices/carriers for the formation of LUT ASDs (good physical stability of LUT, even after 21 days of storage). **DSC** analysis confirmed that ASDs of LUT with PVP K90 and PVP VA64 are fully miscible (a single T_g_). **XRPD** analysis confirmed the complete alteration of LUT’s crystalline structure to an amorphous form for all obtained SDs (halo” effect in diffractograms). **FT-IR** analysis confirmed the formation of molecular interactions between LUT and PVP (between –OH group of LUT and PVP’s C=O group).

Alshehri et al. [81] studied solid dispersions (SDs) of LUT with PEG4000 prepared by fusion, solvent evaporation, and microwave irradiation. **XRPD** analysis confirmed the complete alteration of LUT’s crystalline structure to an amorphous form for all obtained SDs (disappearance of the characteristic peaks of LUT). This was also confirmed by **DSC** analysis (disappearance of melting point for LUT observed at 338.4 °C in pure compound). **SEM** images confirmed that particles of LUT have a crystal shape and that PEG 4000 has irregularly shaped particles with smooth surfaces. In contrast, all SDs had particles with an irregular shape and a rough and irregular surface. **FT-IR** analysis provided evidence that LUT’s characteristic peaks in SDs displayed no significant variation compared to the pure LUT.

**Magnolol (MAG)**

Cao et al. [82] studied solid dispersions (SDs) of MAG with HPMCAS (3 grades: HF, MF, and LF) prepared by antisolvent coprecipitation. **XRPD** analysis confirmed the complete alteration of MAG’s crystalline structure to an amorphous form for all obtained SDs (halo” effect in diffractograms). This was also confirmed by **DSC** analysis (disappearance of melting point for MAG observed at 102 °C in pure compound). **SEM** images confirmed that particles of MAG have an irregular block shape and that HPMCAS has an irregular particle shape with a porous structure. In contrast, in MAG-HPMCAS images, crystalline particles of MAG were detected. **FT-IR** analysis confirmed the shift of the characteristic MAG bands (1635 cm^−1^ (C=C stretching), 1496 cm^−1^ (C=C aromatic stretching), and 1226 cm^−1^ (phenolic hydroxyl stretching)) and the HPMCAS band (2940 cm^−1^ (–OCH_3_ stretching)). **Stability studies** confirmed that MAG-HPMCAS(LF) SDs (2:8 *w*/*w*) remained amorphous for 270 days.

Zhao et al. [83] studied solid dispersions (SDs) of MAG with PlasdoneS-630 (PS-630) prepared by solvent volatilization. **XRPD** analysis confirmed the complete alteration of MAG’s crystalline structure to an amorphous form for all obtained SDs (disappearance of Bragg peaks characteristic of MAG). **FT-IR** analysis confirmed hydrogen-bonding interactions between MAG and PS-630 (complete disappearance of the characteristic peak at 3157.6 cm^−1^ corresponding to the –OH bond).

Liu et al. [84] studied solid dispersion (SD) of MAG with PVP30 prepared by solvent evaporation. **XRPD** analysis confirmed the complete alteration of MAG’s crystalline structure to an amorphous form for the obtained SD (halo” effect in the diffractogram).

**Myricetin (MYR)**

Rosiak et al. [85] developed ASDs of MYR using PVP K30. The ASDs were prepared using combined solvent evaporation and freeze-drying methods. Screening studies demonstrated that complete amorphization was achieved only at higher polymer loadings, with optimal formulations obtained at 1:8 and 1:9 MYR:PVP (*w*/*w*) ratios. The amorphous nature and miscibility of MYR in the polymer matrix were confirmed by XRPD and DSC analyses. **XRPD** patterns of the ASDs showed the disappearance of the sharp Bragg peaks characteristic of crystalline MYR and the presence of a broad halo pattern, indicating loss of long-range order. **DSC** thermograms revealed the absence of the MYR melting endotherm (~364–368 °C) and the presence of a single glass transition temperature (Tg ≈ 178–180 °C), confirming molecule-level dispersion and the formation of a single-phase amorphous system. The presence of a single Tg further indicated full miscibility and reduced risk of phase separation during storage. FT-IR provided strong evidence of specific intermolecular interactions stabilizing amorphous MYR. Significant band shifts, as well as broadening and disappearance of characteristic MYR vibrations, were observed in the ASDs compared with crystalline MYR and physical mixtures. In particular, shifts of the PVP carbonyl stretching band (~1665 cm^−1^) to lower wavenumbers and broadening of the MYR hydroxyl stretching region (2800–3600 cm^−1^) indicated the formation of intermolecular hydrogen bonds between MYR phenolic –OH groups and the carbonyl groups of PVP30. These interactions were further supported by molecular docking studies, confirming hydrogen bonding as the dominant stabilization mechanism in the ASDs. **Stability studies** confirmed that the optimized MYR-PVP 1:9 *w*/*w* ASD remained physically stable for at least two months under ambient conditions, supporting its suitability as a promising delivery system for MYR.

Zhang et al. [86] investigated ASDs of MYR with three pharmaceutically relevant polymers: PVP, HPMC and PEG were selected as carriers to compare their ability to inhibit MYR crystallization and stabilize the amorphous state. The solid dispersions were prepared using a solvent-based method combining rotary evaporation and freeze drying. MYR and polymer excipients (1:9 *w*/*w*) were co-dissolved in an ethanol/dichloromethane mixture, followed by solvent removal, vacuum drying, lyophilization, and cryogenic milling to obtain homogeneous amorphous powders. The solid-state properties of MYR in the dispersions were characterized using DSC and XRPD. **DSC** thermograms showed the disappearance of the sharp melting endotherm of crystalline MYR (~314 °C) in all solid dispersions, indicating effective amorphization and good drug–polymer compatibility. **XRPD** analysis further confirmed the loss of long-range crystalline order, as the intense diffraction peaks of MYR were replaced by broad, amorphous halos. Quantitative crystallinity analysis revealed that the PVP-based system exhibited the strongest inhibition of crystallization, reducing MYR crystallinity from ~95% to below 3%, followed by HPMC and PEG systems. **FT-IR** demonstrated the presence of specific intermolecular interactions between MYR and polymer excipients. In the solid dispersions, the characteristic phenolic hydroxyl stretching bands of MYR (∼3417 cm^−1^ and 3285 cm^−1^) became broadened or disappeared compared with crystalline MYR and physical mixtures, indicating the formation of hydrogen bonds between MYR hydroxyl groups and the carbonyl or ether functionalities of the polymers. These interactions were most pronounced in the MYR/PVP system, consistent with its superior crystallization inhibition. To rationalize the experimental findings, **molecular dynamics** and **quantum mechanical simulations** were employed to quantify miscibility and interaction strength. Calculated Flory–Huggins interaction parameters (χ), mixing energies (ΔE_mix_), mean-square displacement (MSD), and binding energies (E_binding_) consistently ranked polymer effectiveness in the order of PVP > HPMC > PEG, indicating the strongest MYR-polymer affinity and lowest molecular mobility in the PVP-based dispersion. Radial distribution function analysis further confirmed the formation of multiple types of intermolecular hydrogen bonds, with the highest bond strength and molar concentration observed for MYR–PVP interactions.

Mureşan-Pop et al. [87] studied ASDs of MYR with PVP K30 prepared by spray drying. **XRPD** diffractograms for MYR-PVP SD1 10:90 *w*/*w* and SD2 50:50 *w*/*w* had only a few Bragg peaks, with weak intensities confirming a prevalent amorphous state, whereas the 80:20 SD had the highest MYR amount, confirming both an amorphous and crystalline structure. **DSC** analysis of the obtained SDs suggested interactions between MYR and PVP (based on the change in the melting point of MYR with PVP content). **FT-IR** analysis confirmed that intermolecular hydrogen-bonding interactions occurred between MYR and PVP in SDs. A **stability study** (at 40 °C and 75% relative humidity) confirmed that both SD-1 and SD-2 were amorphous after two months under stress conditions.

### 3.2. Patent Landscape

Industrial interest in ASD-based stabilization of polyphenols is evident in the patent literature.

Patent WO 2019/018774 A1 [129] describes polymer-stabilized ASDs of epigallocatechin gallate. **XRPD** and **DSC** confirmed the amorphous state. The reported systems employ hydrophilic polymers, including HPMC-AS, HPMC-P, Soluplus^®^, and cellulose acetate, as stabilizing matrices.

US 11,672,770 B2 [130] addresses orally administered compositions containing amorphous curcumin stabilized with hydrophilic polymers such as HPMC and HPMC-AS. Amorphization was verified by **XRPD** and **DSC** analyses. The systems were prepared via melt processing, and the patent further reports improved oral performance of the amorphous formulation in an animal model.

WO 2012/049253 A1 [131] and EP2627195B1 [132] describes melt-extruded solid dispersions of curcuminoids in hydrophilic polymer matrices based on HPMC. The amorphous state was verified by **XRPD** analysis, as evidenced by the absence of characteristic crystalline diffraction peaks, with residual crystallinity reported to be below the detection limit in selected formulations.

EP 4 106 725 B1 [133] reports amorphous solid dispersions of quercetin formulated with polyvinylpyrrolidone (PVP K17) and an alkaline component. The amorphous state was confirmed using **XRPD** and **DSC**, demonstrating the absence of crystalline reflections and melting transitions characteristic of quercetin. The system was obtained through solvent-based processing, including spray drying, yielding a polymer-stabilized quercetin solid dispersion.

## 4. Carriers Used in ASDs of Polyphenols

The selection of carriers in amorphous solid dispersions of polyphenols is one of the key factors determining the success of a formulation. The physicochemical properties of the carrier, such as its glass transition temperature, polarity, hydrogen-bonding capacity, hydrophilic–hydrophobic character, and behavior in an aqueous environment, determine both the efficiency of amorphization and the long-term stability of the system [134,135,136]. Moreover, it has been confirmed that the appropriate selection of carriers makes it possible to achieve improved solubility, dissolution rate, and bioavailability. As a result, this translates into improved biological properties of polyphenols.

### 4.1. Cyclodextrins and Their Derivatives

Cyclodextrins are characterized by the presence of a hydrophobic cavity and a hydrophilic outer surface, which predisposes them to enhance the solubility of phenolic compounds through the formation of host–guest interactions [137,138,139,140,141]. In the context of ASDs, however, their ability to stabilize the amorphous state is limited, as their mechanism of action is based mainly on complexation rather than on the formation of a continuous, amorphous polymeric matrix [73,142,143]. Hydroxypropyl derivatives of β-cyclodextrin (HP-β-CD) exhibit greater structural flexibility and higher aqueous solubility, favoring the formation of non-crystalline systems and improving the dissolution of polyphenols [78,138,139]. Cyclodextrins can be treated as functional excipients rather than as primary carriers responsible for long-term stabilization of ASDs.

### 4.2. Amino Acids and Low-Molecular-Weight Compounds

Amino acids and other low-molecular-weight compounds are widely used as co-formers in co-amorphous polyphenol systems [34,40,100]. Amino acids exhibit properties distinct from those of conventional polymers. Their low molecular weight and ability to form strong ionic and hydrogen-bonding interactions with polyphenols promote efficient stabilization of the amorphous state using significantly lower amounts of carrier. Garbiec et al. [33,34,144] indicate that basic amino acids, such as arginine and lysine, are particularly effective in stabilizing phenolic compounds containing phenolic and carbonyl functional groups, which is attributed to the formation of an ordered network of intermolecular interactions. Co-amorphous systems often exhibit higher physical stability than conventional polymer-based ASD formulations [100].

### 4.3. Polyvinylpyrrolidone and Its Derivatives

Polyvinylpyrrolidone and its derivatives are among the most versatile polymers used in ASDs, which results from their high polarity, large number of hydrogen bond acceptor groups, and relatively high glass transition temperature. These properties promote both efficient amorphization of active pharmaceutical ingredients and inhibition of their recrystallization during storage. The literature confirms that hydrogen bonding interactions between the –C=O groups of polyvinylpyrrolidone (PVP) and proton donors present in active compounds play a key role in stabilizing the amorphous state [18,31,64,96,144,145,146]. In studies on ASDs of polyphenols, PVP grades such as K29/32, K30, and K90 have been most frequently employed (see Table 1). Different molecular weight fractions of PVP exhibit distinct stabilizing properties. The effect of PVP’s molecular weight on ASD stability is not unequivocal and depends on the specific system; both excessively low and very high molecular weights may adversely affect crystallization inhibition [134,136,147]. Higher molecular weight generally contributes to reduced molecular mobility; however, it may also lead to increased free volume within the polymer matrix. Due to the high hygroscopicity of PVP, moisture uptake can negatively affect the stability of the resulting ASDs [146,148,149]. Vinylpyrrolidone–vinyl acetate copolymers such as PVP VA64 exhibit lower hygroscopicity compared to PVP and good processability, making them particularly attractive for technologies such as HME [41,97,102,150,151]. The presence of vinyl acetate segments reduces the strength of hydrogen-bonding interactions with the active compound relative to PVP, which may result in slightly weaker crystallization inhibition; however, it simultaneously improves the stability of ASDs under humid conditions.

### 4.4. HPMC and Its Derivatives

Cellulose derivatives constitute one of the most important and extensively studied groups of carriers used in ASDs. Their widespread application in ASD formulations arises from favorable physicochemical properties, such as a high glass transition temperature, good compatibility with a wide range of active pharmaceutical ingredients, and the ability to effectively inhibit crystallization both in the solid state and upon contact with aqueous media. In the case of polyphenols, which are characterized by a high number of hydroxyl groups and a strong tendency to crystallize, cellulose derivatives enable the formation of stable amorphous systems with improved biopharmaceutical performance [51,52,60,61,62,127]. HPMC is a widely used ASD carrier due to its good compatibility with numerous active compounds and its ability to form stable amorphous matrices. It exhibits high efficiency in inhibiting crystallization in the solid state; however, its high viscosity limits its applicability in conventional HME. Consequently, HPMC is more frequently employed in solvent-based processes, including spray drying [110], solvent evaporation [70,99,121,122], and freeze drying [86,108]. HPMCAS is one of the most effective polymers used in ASD formulations, owing to its amphiphilic character, low hygroscopicity, and the presence of acidic functional groups that enable strong interactions with active compounds. It is capable of maintaining a supersaturated state in solution, effectively inhibiting crystallization after dissolution. These properties make HPMCAS particularly suitable for formulations of compounds with very low aqueous solubility [61,68,92,105,106]. HPMCP, as a cellulose derivative containing phthalate groups, exhibits the ability to form strong ionic interactions with basic active pharmaceutical ingredients. Such interactions can significantly enhance the physical stability of ASDs, even under high drug loading. At the same time, HPMCP functions as an enteric polymer, enabling modification of the drug-release profile [69,110]. Hydroxypropyl cellulose (HPC-SL and HPC-SSL) is characterized by good thermal processability and low viscosity, which makes it suitable for application in HME. HPC can effectively enhance the dissolution rate of ASDs; however, its ability to provide long-term amorphous stabilization may be lower compared to HPMCAS [136]. More complex cellulose derivatives such as CAAdP, CMCAB, and CASub contain both hydrophilic and hydrophobic side groups. This molecular architecture confers a pronounced amphiphilic character and promotes the formation of stable amorphous matrices [61,68,92,105,106]. These polymers combine the advantages of classical cellulose derivatives with properties characteristic of next-generation polymers, demonstrating high effectiveness in crystallization inhibition and good supersaturation stabilization capability. Nevertheless, their application in polyphenol formulations remains limited, and the number of experimental studies is significantly lower compared to HPMC or HPMCAS, indicating substantial potential for further research in this area [136].

### 4.5. Acrylic Polymers (Eudragit and Derivatives)

Methacrylate polymers are characterized by distinctly differentiated pH-dependent dissolution properties, which enable the design of amorphous solid dispersions with targeted sites and controlled release profiles. Cationic polymers from the Eudragit E group exhibit solubility in acidic environments and the ability to form strong ionic interactions with acidic compounds [152,153,154]. The literature [32,41,56,73] indicates that Eudragit E PO can significantly enhance the solubility and bioavailability of active substances through the formation of ionic complexes and micellar structures in solution, making it particularly suitable for oral ASD formulations. Anionic methacrylate polymers such as Eudragit L, L100, L100-55, and S100 are primarily employed in the design of ASDs with pH-dependent and delayed drug release. Eudragit L and S have been shown to effectively stabilize the amorphous state of the active substance and promote intestinal release. Furthermore, these polymers have demonstrated the ability to inhibit crystallization both in the solid state and under supersaturated conditions.

### 4.6. Surfactants and Poloxamers

Surfactants and poloxamers are used in ASDs primarily as functional excipients that support the dissolution process, improve wettability, and stabilize the supersaturated state following the release of polyphenols from the polymer matrix [37,38,42,47,72,110]. In contrast to conventional polymers with high glass transition temperatures, their role in ASD formulations is mainly associated with modulation of system behavior during dissolution rather than with long-term stabilization of the amorphous state in the solid phase [155,156]. Poloxamers are nonionic, amphiphilic triblock copolymers of the PEO-PPO-PEO type that exhibit the ability to self-assemble in aqueous solutions and form micellar structures [157,158,159]. In ASDs of polyphenols, poloxamers are most commonly applied as components of binary [37,38,72] or ternary [42] systems, where they provide solubilization and enhance particle wettability upon dissolution of the dispersion. The presence of poloxamers can significantly increase the dissolution rate and the maximum concentration of polyphenols in solution by facilitating the dispersion of amorphous molecules and stabilizing supersaturation. The application of poloxamers is limited, among other factors, by their low glass transition temperature. This property promotes increased molecular mobility and may lead to accelerated recrystallization of the active compound in the solid state, particularly in combination with high poloxamer content in the formulation. For this reason, poloxamers are most often used in combination with high-Tg polymers, e.g., PVP [42].

### 4.7. Polyethylene Glycol (PEG)

PEG, a polymer of ethylene oxide with a broad molecular weight range (200–300,000 g/mol), is widely used as a carrier in solid dispersions and ASDs. Owing to its relatively low melting temperature (55–68 °C) and high solubility in aqueous and volatile organic media, PEG can be processed using both solvent-based and melt-based techniques. These physicochemical properties facilitate its application in the preparation of solid dispersions and ASDs, particularly for poorly water-soluble, low-melting-point drug compounds [160]. PEG 4000 and PEG 6000 are most frequently employed in ASDs of polyphenols [53,70,81,93,98,102,112].

## 5. Preparation Methods of Amorphous Solid Dispersions

The preparation method plays a crucial role in determining the physicochemical properties, stability, and performance of ASDs. Various processing techniques have been employed to obtain amorphous polyphenol-based systems, each characterized by distinct mechanisms of amorphization and formulation constraints. This section provides an overview of the most commonly reported preparation methods used for ASDs of polyphenols.

### 5.1. Solvent Evaporation

Solvent evaporation methods involve dissolving both the active substance and the carrier(s) in a common solvent or solvent mixture, followed by removal of the solvent. During solvent elimination, the components of the system become increasingly concentrated, which facilitates the formation of a homogeneous solid phase and may result in the generation of an amorphous product. According to the literature, solvent evaporation techniques can be classified into four main categories based on the conditions applied for solvent removal: elevated temperature at atmospheric pressure, elevated temperature under reduced pressure, freeze drying, and processing using supercritical fluids [33,161,162].

### 5.2. Spray Drying

Spray drying is a technique that enables the production of free-flowing powders with controlled particle sizes ranging from the nanometer to micrometer scale from liquid or semi-solid feed materials. In this process, the active substance and carrier are dissolved or suspended in a suitable solvent system—most commonly, methanol, acetone, or their mixtures. Spray drying is frequently applied during the early stages of drug development, when only limited amounts of the active substance are available.

The spray dryer consists of a drying chamber, into which the drug solution or suspension is introduced through a nozzle, where atomization occurs. During spraying, the generated droplets come into contact with a stream of hot drying gas—most commonly, air. Owing to rapid solvent evaporation, which typically occurs within seconds, spray drying is particularly suitable for the preparation of ASD systems containing compounds with limited thermal stability. Nevertheless, strict control of process parameters, including the inlet temperature and chamber humidity, is required, as these factors may significantly influence the amorphization outcome. Spray drying is considered a scalable technique and is therefore widely used for the preparation of amorphous solid dispersions [163,164].

### 5.3. Hot-Melt Extrusion (HME)

Hot-melt extrusion is a processing technology originally developed in the food industry, where it is used for the mixing, cooking, kneading, and shaping of materials, as well as to reduce microbial contamination and enzyme activity [165]. In pharmaceutical applications, HME is employed as a solvent-free technique that enables intensive mixing of the active substance with a polymeric carrier, resulting in good content uniformity. The absence of organic solvents represents a significant advantage of HME compared to solvent-based techniques such as spray drying.

The process is carried out using a hot-melt extruder, which enables melting, mixing, and homogenization of the drug and carrier under controlled conditions. The extruder is equipped with temperature-controlled heating zones and rotating screws, allowing for precise regulation of temperature, shear forces, and material flow. During processing, drug particles are dispersed within the molten polymer matrix, leading to the formation of a homogeneous dispersion. As reported in the literature, HME enables intimate mixing of the drug and polymer, which may result in molecule-level dispersion within the carrier matrix [120,166,167].

### 5.4. Freeze Drying/Lyophilization

Freeze drying, also referred to as lyophilization, is a process in which a solution or suspension is first cooled until the solvent is completely solidified and subsequently removed by sublimation under reduced pressure. During this process, the frozen solvent is converted directly from the solid to the vapor phase at a low temperature, allowing for the removal of up to approximately 99% of the solvent content. Compared to spray drying, freeze drying is generally considered a less energy-intensive and more economically favorable technique for laboratory-scale production. However, its application in the preparation of amorphous solid dispersions is primarily limited to systems employing water-soluble polymeric carriers, as the process is not suitable for polymers that require organic solvents for dissolution [168,169].

### 5.5. Milling

Milling, also referred to as comminution or grinding, is a mechanical process used to reduce particle size through the application of mechanical energy. Ball milling is often regarded as a green chemistry technique [170,171]. Depending on the mode of energy transfer, milling devices can be classified as ball mills, shear-action mills, and impact (shock-action) mills. Regardless of the type of equipment employed, the primary outcome of milling is a reduction in particle size. The process is typically conducted without the use of organic solvents and, when properly controlled, without intentional thermal input. However, as reported in the literature, unintended effects may occur during milling, including changes in crystallinity [172], temperature increases [173], and alterations in chemical stability [174], which may become evident during subsequent storage after grinding. In addition, milling has been shown to induce aggregation of fine particles, the generation of electrostatic charges, and enhanced solid-state reactivity [175]. During the grinding process, particles are subjected to intense mechanical stresses that can disrupt the crystal lattice, leading to partial or complete amorphization of the substance. Despite confirmation that milling can produce amorphous pharmaceutical compounds, this technique is not widely applied for the preparation of ASD systems. One of the limiting factors is the challenge associated with process scale-up [176,177]. Moreover, heat generated during milling may adversely affect thermolabile substances [178]. A specific variant of this technique is co-milling, in which the active substance is milled in the presence of hydrophilic excipients. This approach has been reported to improve milling efficiency and represents a simple and economical strategy to enhance the processing of poorly soluble compounds [179].

### 5.6. Cryo-Grinding/Cryo-Milling

In cryo-milling, the crystal lattice is mechanically disrupted in a manner similar to conventional milling. Compared to standard milling, this technique is characterized by improved process efficiency, which results from the use of cryogenic conditions typically achieved by cooling with liquid nitrogen. At such low temperatures, materials become more brittle, facilitating particle size reduction and micronization. Owing to the absence of significant thermal stress, cryo-milling can be successfully applied to thermolabile substances [178].

### 5.7. Supercritical Carbon Dioxide (scCO_2_)

The supercritical carbon dioxide method involves the preparation of ASDs under supercritical conditions, in which CO_2_ exhibits both gaseous and liquid properties. During the process, the active compound and the polymeric carrier are exposed to supercritical carbon dioxide at elevated pressure and moderate temperature, leading to plasticization of the system, a reduction in the glass transition temperature, and facilitation of molecular reorganization. After exposure to supercritical carbon dioxide and subsequent decompression, carbon dioxide is rapidly removed from the system, promoting the stabilization of the disordered structure and the formation of an amorphous phase. This method enables the preparation of ASDs under thermally mild conditions without the use of organic solvents, making it particularly suitable for thermolabile substances [36,180,181].

### 5.8. Quench Cooling

The quench cooling method involves the attainment of ASDs by melting the active substance, either alone or in the presence of a polymer (or an excipient), followed by its rapid cooling to inhibit the crystallization process. During the process, the components of the system are heated above the melting point, which leads to the destruction of the ordered crystal structure. Then, the alloy is quickly cooled, most often using liquid nitrogen or intensive contact cooling, which results in the “freezing” of the disordered molecular system and the formation of an amorphous phase. Due to the need to use elevated temperatures, this method has limited application in the case of thermolabile substances and systems susceptible to thermal degradation. The obtained material can be further crushed and stored under conditions that reduce the risk of recrystallization [65,182,183].

### 5.9. Electrospinning

Electrospinning is a method used to produce ASDs that involves the application of an electrostatic field to convert a polymer solution or melt containing an active compound into fine fibers [184]. During the process, a polymer solution with a dissolved polyphenol is fed through a capillary needle, at the tip of which a so-called Taylor cone [185,186] is formed under the influence of high voltage. When the critical value of electrostatic forces is exceeded, a thin liquid jet is ejected, which undergoes stretching and rapid solidification as a result of intensive solvent evaporation. The resulting fibers have micro- or nanometric diameters, with the active compound uniformly dispersed within the polymer matrix, typically in an amorphous state [71,142,185,186,187,188,189]. Electrospinning can be performed in various process configurations, including single- and multi-stream systems.

## 6. Solid-State Characterization Techniques Used to Confirm Amorphization

The in-depth assessment of ASDs necessitates the utilization of a spectrum of analytical techniques. Thus comprehensive analysis encompasses an exploration of their physical, chemical, and structural attributes to gain a nuanced understanding of their behavior and potential applications. Table 2 summarizes diverse analytical methods used during the characterization of ASDs.

## 7. Challenges and Future Perspectives

The therapeutic potential of polyphenols has been extensively documented over the decades. The literature reviewed here demonstrates the rapid expansion of the amorphization strategy for polyphenolic compounds. Despite numerous successes, several critical challenges remain.

The most fundamental issue is the physical stability of the amorphous state. From a thermodynamic perspective, the amorphous form is inherently unstable relative to its crystalline counterpart. Consequently, ASD systems may undergo recrystallization over time, even when initially confirmed as fully amorphous. Such transformations may occur during storage or downstream processing or under physiological conditions, ultimately compromising solubility enhancement and limiting further formulation development. Therefore, long-term stability assessment under relevant environmental conditions remains essential [192,193].

Another key challenge concerns the generation and maintenance of supersaturation. Although ASDs can produce significantly elevated apparent solubility, the supersaturated state is metastable and may rapidly collapse due to nucleation and crystal growth. Under physiological or biorelevant conditions, the duration of supersaturation may be insufficient to enable adequate drug absorption, particularly for compounds with limited permeability or rapid precipitation kinetics. This highlights the importance of polymer selection not only for amorphization efficiency but also for precipitation inhibition and maintenance of supersaturation [194]. The rational selection of preparation methods and carrier polymers remains a complex, compound-specific task. Extensive experimental screening is often required to identify optimal combinations that ensure efficient amorphization and long-term stabilization. In this context, in vivo studies are particularly valuable, as they provide direct evidence of improved bioavailability in animal models and allow for verification of in vitro–in vivo correlations [195,196]. A stronger emphasis on the mechanistic understanding of molecular interactions between polyphenols and polymer matrices may further support predictive formulation design [197].

Economic feasibility represents an additional, often underestimated factor. The cost of pharmaceutical-grade polymers and the scalability of amorphization techniques can significantly influence the translational potential of ASD systems. Some methods, such as hot-melt extrusion, may be energy-intensive, whereas others, including ball milling or electrospinning, may present scalability limitations. From a green chemistry perspective, solvent-free approaches (e.g., hot-melt extrusion, ball milling, supercritical fluid techniques, or KinetiSol processing) are increasingly favored, as they reduce environmental impact and eliminate the risk of residual organic solvents [198]. Nevertheless, each method must be evaluated individually, not only in terms of amorphization efficiency but also in terms of the risk of compound degradation induced by elevated temperatures, mechanical stress, or shear forces. The growing body of literature in this field contributes valuable process knowledge that facilitates a more application-oriented approach to ASD development. Improved understanding of critical formulation parameters may shorten development timelines and reduce empirical screening efforts. The integration of advanced analytical techniques, computational modeling, and predictive tools for glass-forming ability and recrystallization risk may further accelerate progress [199,200].

An emerging and particularly interesting direction is the adaptation of ASDs for non-oral routes of administration. For example, wound environments—characterized by high moisture levels—pose a significant risk of recrystallization, yet they also offer opportunities for local delivery of poorly soluble polyphenols with antioxidant or anti-inflammatory properties. Expanding ASD technology beyond traditional oral dosage forms may therefore open new therapeutic avenues [198,201].

In summary, while significant challenges remain—particularly regarding stability, supersaturation maintenance, scalability, and cost-effectiveness—the continuous evolution of formulation science suggests that ASDs will play an increasingly important role in enabling the pharmaceutical development of polyphenols. The expanding methodological toolbox and growing mechanistic insight support a future in which amorphization strategies broaden the range of viable polyphenolic product candidates and enhance their translational potential.

## 8. Summary and Scope of Part II

The first part of this review has focused on formulation-related aspects of amorphous solid dispersions of polyphenols, including the types of investigated compounds, polymeric carriers, preparation methods, and solid-state characterization techniques used to confirm amorphization. The presented analysis highlights the diversity of formulation strategies applied to polyphenols and emphasizes the critical role of carrier selection and processing approaches in achieving physically stable amorphous systems.

Part II of this review will address the biopharmaceutical and biological implications of amorphous solid dispersions of polyphenols. Particular emphasis will be placed on the impact of amorphization on solubility enhancement, dissolution behavior, maintenance of supersaturation, and oral bioavailability. In addition, Part II will discuss the influence of carrier selection on the release behavior of polyphenols from amorphous matrices, including potential pH-dependent dissolution effects. Furthermore, reported in vitro and in vivo studies will be examined to evaluate improvements in antioxidant, anti-inflammatory, and other biological activities. Finally, safety considerations and translational aspects relevant to functional food and pharmaceutical applications will also be discussed.

## Figures and Tables

**Table 1:** Reported polyphenol-based ASDs: compounds, carriers, preparation methods, and solid-state characterization.

Carrier	Method	Identification	References
** APIGENIN **			
Soluplus	scCO_2_	XRPD FT-IR	[36]
Pluronic F-68 Pluronic F-127	Ball milling	XRPD TG DSC SEM FT-IR	[37]
Pluronic F-127	Spray drying	XRPD DSC SEM FT-IR	[38]
** BAICALEIN **			
Nicotinamide	Solvent evaporation	XRPD DSC SEM FT-IR PLM	[39]
Histidine	Solvent evaporation	XRPD DSC FT-IR	[40]
PVP VA 64 Eudragit E PO	HME	XRPD DSC FT-IR	[41]
** BUTEIN **			
PVP K30 PVP K30/poloxamer 407	Solvent evaporation	DLS XRPD DSC	[42]
** CHRYSIN **			
Plasdone^®^ S630	Solvent evaporation	XRPD	[43]
Brij^®^L4	Solvent evaporation	XRPD DSC SEM	[44]
** CURCUMIN **			
Eudragit E PO	HME	XRPD DSC FT-IR	[45]
Eudragit/PVP, Eudragit/HPMC	Solvent evaporation	XRPD FT-IR	[46]
HPC SDS	Vibrational ball milling	XRPD DSC FT-IR	[47]
Eudragit RSPO Eudragit RLPO	HME	DSC XRPD	[48]
COS	Ball milling	DSC XRPD	[49]
PVP K30	Solvent evaporation	DSC IR XRPD Raman SEM NMR	[50]
HPMC	Solution mixing method	DSC SEM	[51]
HPMC Eudragit E100	Solvent evaporation	PLM	[52]
HPMC E5 Eudragit E100	Solvent evaporation	Raman Raman imaging IR Fluorescence DLS	
PEG 6000 PVP K30 Eudragit E PO PVP K30/Eudragit E PO Eudragit E PO + HPMC E50	Solvent evaporation	Raman Raman imaging IR	[53]
PVP K30	Solvent evaporation	DSC XRPD	[54]
α-glucosyl stevia PVP K30	Freeze drying	DSC XRPD	[55]
Eudragit E PO	Solvent evaporation	DSC XRPD	[56]
Eudragit E PO	Solution mixing	DSC XRPD FT-IR ^1^H NMR	[57]
Eudragit E PO	Spray drying Rotary evaporation	DSC XRPD	[58]
HPMC/lecithin/isomalt	Hot-melt extrusion	XRPD DSC	[59]
Eudragit E100 HPMC E5	Solvent evaporation Cryo-milling	XRPD DSC UV-Vis IR	[60]
HPMCAS CMCAB CAAdP	Spray drying	XRPD DSC FT-IR	[61]
HPMC-AS	Freeze drying	XRPD DSC SEM PLM TEM	[62]
PVP	Spray drying	DSC XRPD	[63]
**CURCUMIN-HESPERETIN**			
PVP K30/phosphatidylcholine	HME	DSC XRPD FT-IR	[64]
**CURCUMIN-MAGNOLOL**			
HPMC HPC PVP K30	Quench cooling	XRPD DSC Raman FT-IR NMR	[65]
** CURCUMIN-PIPERINE **			
PVP VA64	HME	DSC XRPD FT-IR	[31]
** DAIDZEIN **			
PVP K90	Spray drying	XRPD DSC SEM	[66]
** DIOSMIN **			
Soluplus	Spray drying	XRPD DSC SEM FT-IR	[67]
** ELLAGIC ACID **			
CMCAB CAAdP HPMCAS PVP CAAdP/PVP	Spray drying Co-precipitation Solvent evaporation	XRPD MDSC IR NMR	[68]
** EPIGALLOCATECHIN GALLATE **			
PMCAS HPMCP Soluplus Cellulose acetate	Freeze drying	XRPD SEM PLM TG DSC	[69]
**FERULIC ACID** * (complete amorphization was not obtained)			
HPMC Soluplus PVP VA64 PVP K30 PEG 6000	Kneading Solvent evaporation	XRPD FT-IR SEM DSC	[70]
PVA PVA/PVP K10	Electrospinning	XRPD	[71]
PVP K30 PEG 6000 Poloxamer 188	Spray drying	SEM XRPD TGA IR	[72]
**FISETIN**			
Eudragit EL100 Eudragit EPO Eudragit EL100/HPβCD Eudragit EPO/HPβCD	Ball milling	DSC XRPD FT-IR	[73]
P(VP-co-VAc)	Supercritical carbon dioxide	XRPD FT-IR	[74]
Polymers based on cyclodextrins Poly-aβ-CD Poly- aγ-CD Poly-aβγ-CD Poly-methyl-β-CD	Spray drying	TG DSC SEM NMR FT-IR	[75]
**GENISTEIN**			
CAG CP-HPC HPMCAS	Solvent evaporation	XRPD DSC FT-IR	[76]
Lysine Arginine	Ball milling	XRPD TG DSC FT-IR	[34]
PVP K-30	Solvent evaporation	XRPD DSC SEM FT-IR	[77]
** HESPERETIN **			
HP-β-CD	Solvent evaporation	XRPD DSC FT-IR	[78]
** HESPERETIN-PIPERINE **			
PVP VA64	Ball milling	XRPD DSC FT-IR	[79]
** HESPERIDIN **			
Alginate sodium HPMC Soluplus	Ball milling	XRPD DSC FT-IR	[19]
HP-β-CD	Solvent evaporation	XRPD DSC FT-IR	[78]
** KAEMPFEROL **			
Eudragit E PO Eudragit EL100 Eudragit EL100-55	Ball milling	XRPD DSC FT-IR	[32]
** LUTEOLIN **			
PVP K90 PVP VA64 Soluplus HPC-SL HPMCAS Eudragit E PO	Film casting	PLM XRPD DSC FT-IR	[80]
PEG 4000	Fusion Solvent evaporation Microwave irradiation	XRPD DSC SEM NMR FT-IR	[81]
** MAGNOLOL **			
HPMCAS	Antisolvent coprecipitation	XRPD DSC SEM FT-IR	[82]
PlasdoneS-630 PVP VA64 PVP K30 Soluplus	Solvent volatilization	XRPD FT-IR	[83]
PVP K30	Solvent evaporation	XRPD	[84]
** MYRICETIN **			
PVP K30	Solvent evaporation + freeze drying	DSC XRPD FT-IR	[85]
HPMC PEG PVP	Combination of rotary evaporation and freeze drying	DSC XRPD FT-IR	[86]
PVP K30	Spray drying	DSC XRPD FT-IR	[87]
** NARINGENIN **			
PVP K25 PGFE	HME	DSC XRPD	[88]
Poloxamer 188/Neusilin US2	HME	DSC XRPD HSM IR	[89]
Soluplus	Solvent evaporation Kneading	DSC XRPD FT-IR	[90]
Eudragit EL100 PVP K29/32 HPMCAS HPMC CMCABP PAA		XRPD IR	[91]
CAAdP CMCAB HPMCAS PVP K29/32	Spray drying	XRPD DSC TG FT-IR NMR	[92]
PVP K30 PEG 4000	Solvent evaporation	XRPD SEM TEM IR	[93]
** NOBILETIN **			
HPC-SSL	Wet milling Freeze drying	XRPD DSC SEM PLM	[94,95]
** PTEROSTILBENE **			
PVP K30 PVP VA64	Ball milling	XRPD DSC FT-IR	[96]
Soluplus	Ball milling	XRPD DSC SEM FT-IR	[18]
** POLYDATIN **			
PVP VA64 Soluplus Kollicoat IR	HME	XRPD DSC FT-IR	[97]
** QUERCETIN **			
CAG CP-HPC HPMCAS (reference polymer)	Spray drying	XRPD DSC PLM	[76]
PEG 1000 PEG 4000 PEG 6000	Melt mixing	XRPD DSC FT-IR DVS	[98]
HPMCAS PVP K30 Lysine	Solvent evaporation	DSC	[99]
COS	Ball milling	XRPD DSC FT-IR SEM PLM	[49]
Arginine Glutamic acid Aspartic acid Tryptophan Glycin	Ball milling	XRPD IR	[100]
PVP K30 HPMCAS-HF HPMCAS-MF HPMCAS-LF	Solvent evaporation Co-precipitation	XRPD DSC IR	[101]
PEG 6000 PVP VA64 HPMC E5 Poloxamer 188 (Pluronic F68) Soluplus	HME	XRPD DLS Fluorescence IR Raman	[102]
PVP K30	Spray drying	XRPD IR SEM	[103]
Isoquercitrin α-glucosyl rutin	Solvent evaporation	XRPD DSC SEM	[104]
CCAB HPMCAS CASub/PVP CCAB/PVP	Spray drying	XRD DSC FT-IR	[105]
CMCAB HPMCAS CAAdP PVP K29/32	Spray drying	XRPD DSC GC-MS NMR UV-Vis IR	[106]
β-cyclodextrin PVP (MW 40,000) Pluronic F127	Evaporative precipitation of nanosuspension	SEM	[107]
** RESVERATROL **			
PVP HPMC HPMCAS	Freeze drying	XRPD DSC PLM	[108]
Eudragit E PO/Gelucire 44/14	Freeze drying	SEM	[109]
Soluplus PVPVA HPMCAS HPMCP HPMC Surfactant	Spray drying	DSC	[110]
Eudragit E PO	HME	XRPD DSC SEM PLM	[111]
Eudragit E PO PEG 6000 PVP K30 Soluplus	Solvent method	SEM PLM DSC XRPD NMR FT-IR	[112]
CMCS PVP K29/32	Solvent evaporation	XRPD MDSC SEM FT-IR	[113]
Low-molecular-mass chitosan (poly-D-glucosamine)	Spray drying	XRPD DSC SEM FT-IR	[114]
Soluplus/poloxamer 407	Solvent-based methods	XRPD FT-IR	[115]
Eudragit RS PEG 6000	HME	XRPD DSC SEM FT-IR	[116]
Eudragit E	Spray drying	XRPD SEM	[117]
PEG 6000 Poloxamer F68	Melting	XRPD DSC FT-IR	[118]
Soluplus	Freeze drying	XRPD SEM AFM TEM FT-IR	[119]
Eudragit E PO	Ball milling + HME	XRPD DSC SEM FT-IR	[120]
PVP 29/32 PAA HPMC HPMCAS CMCAB Eudragit EL100	Solvent evaporation	XRPD DSC FT-IR	[121]
PVP 29/32 PAA HPMC HPMCAS CMCAB Eudragit EL100	Solvent evaporation	XRPD PLM UV–Vis Raman	[122]
** RUTIN **			
AEROPERL^®^ 300 Pharma	Solvent evaporation	XRPD DSC SEM LM	[123]
** SINAPIC ACID **			
Arginine Histidine Lysine Tryptophan Proline	Ball milling Solvent evaporation Freeze drying	XRPD TG DSC SEM FT-IR	[33]
** WOGONIN **			
PVP K30	Solvent evaporation	XRPD DSC	[124]

Abbreviations: *—complete amorphization was not obtained, HME—Hot-melt extrusion, DSC—Differential scanning calorimetry, XRPD—X-ray powder diffraction, FT-IR—Fourier-transform infrared spectroscopy, HPMC—Hydroxypropyl methylcellulose, HPC—Hydroxypropyl cellulose, LM—light microscopy, SDS—Sodium dodecyl sulfate, TG—Thermogravimetry, SEM—Scanning electron microscopy, PLM—Polarized light microscopy, DLS—Dynamic light scattering, PVP—Polivinylopirolidon, COS—Amorphous chitosan oligosaccharide, IR—Infrared spectroscopy, HPMCAS—Hydroxypropylmethylcellulose acetate succinate, PEG—Polyethylene glycol, PVP VA—Copolymer of vinylpyrrolidone with vinyl acetate, CCAB—6-carboxycellulose acetate butyrate, CASub—Cellulose acetate suberate, CMCAB—Carboxymethyl cellulose acetate butyrate, CAAdP—Cellulose acetate adipate propionate, GC-MS—Gas chromatography–mass spectrometry, NMR—Nuclear magnetic resonance, UV-Vis—Ultraviolet–visible spectroscopy, HPMCAS—Hydroxypropyl methylcellulose phthalate, CMCS—Carboxymethyl chitosan, MDSC—Modulated differential scanning calorimetry, AFM—Atomic force microscopy, TEM—Transmission electron microscopy, PAA—Polyacrylic acid, HP-β-CD—Hydroxypropyl-β-cyclodextrine. The gray color of a table cell highlights the polyphenol to which a given section of the table applies.

**Table 2:** Techniques for characterizing amorphous solid dispersions (table based on literature [168,190,191]).

		Technique	Information Generated	Property Determined
Main impact on formulation	physical stability	DSC (MDSC)	Tg, heat capacity, and excess properties	Phase miscibility, crystallinity, and impurity
		TGA	Weight loss against temperature	Water content
		PXRD	Diffraction pattern	Phase miscibility and crystallinity
		FTIR/NIR/Raman	IR spectrum and Raman spectrum	Intermolecular interactions (e.g., hydrogen bonding), crystalline and amorphous identification, and phase separation
		Fluorescence spectroscopy	Fluorescence spectrum	Drug–polymer miscibility, phase separation, and drug dissolution behavior in ASDs
		TPS	Electromagnetic spectrum	long-range crystalline lattice vibrations, low-energy torsion, and hydrogen-bonding vibrations
		ssNMR	Spin-lattice relaxation time	Phase miscibility, intermolecular interaction, and molecular mobility
		NQR	Nuclear quadrupole resonance spectrum	Crystalline phase identification, polymorphism, molecular environment of quadrupolar nuclei, and detection of crystallinity and phase transitions
		DVS	Water sorption isotherm	Hygroscopicity
		IGC	Retention volume and dispersive surface free energy	Kinetics of surface relaxation (tendency of surface crystallization)
		PLM	Amorphous crystallinity (birefringence)	Crystalline morphology and size, polymorphic transitions, and crystallization route
		XPS	Surface chemical composition and drug–polymer interaction	Surface elemental composition, chemical state of elements, drug–polymer interactions, and surface composition of amorphous dispersions
Main impact on formulation	dissolution rate	AFM	Surface topography, phase separation, and drug–polymer miscibility	Particle size and miscibility
		SEM (EDS)	Particle morphology and size, rapid measurement of surface crystal, and chemical distribution map (EDS)	Particle size
		Laser diffraction	Particle size distribution	Particle size
		Dissolution	Drug release profile	Solubility and supersaturation level
		BET analysis	BET adsorption profile	Specific surface area
		Densitometer	Density	Porosity and crystallinity
		Viscometer	Viscosity	Viscosity
	bioavailability	Dissolution	Drug release profile	Solubility and supersaturation level
		HPLC	Sample concentration	Drug loading and encapsulation efficiency

Abbreviations: AFM—atomic force microscopy, SEM (EDS)—Scanning electron microscopy (Energy-Dispersive Spectroscopy), BET analysis—Brunauer–Emmett–Teller surface-area analysis, DSC—differential scanning calorimetry, DVS—Dynamic Vapor Sorption, FTIR—Fourier-Transform Infrared Spectroscopy, HPLC—High-pressure liquid chromatography, IGC—Inverse Gas Chromatography, MDSC—Modulated differential scanning calorimetry, NIR—Near-Infrared Spectroscopy, NQR—Nuclear quadrupole resonance, PLM—Polarized light microscopy, PXRD—Powder X-ray diffraction, Raman—Raman spectroscopy, ssNMR—Solid-state nuclear magnetic resonance, TGA—thermogravimetric analysis, TPS—Terahertz pulsed spectroscopy, XPS—X-ray photoelectron spectroscopy.

## Data Availability

The data are contained within the article.

## Abbreviations

The following abbreviations are used in this manuscript:

BCS	Biopharmaceutical classification system
ASDs	Amorphous solid dispersions
HME	Hot-melt extrusion
DSC	Differential scanning calorimetry
XRPD	X-ray powder diffraction
FT-IR	Fourier-transform infrared spectroscopy
HPMC	Hydroxypropyl methylcellulose
HPC	Hydroxypropyl cellulose
SDS	Sodium dodecyl sulfate
TG	Thermogravimetry
SEM	Scanning electron microscopy
PLM	Polarized light microscopy
DLS	Dynamic light scattering
PVP	Polyvinylopirolidon
COS	Amorphous chitosan oligosaccharide
IR	Infrared spectroscopy
HPMCAS	Hydroxypropylmethylcellulose acetate succinate
PEG	Polyethylene glycol
PVP VA	Copolymer of vinylpyrrolidone with vinyl acetate
CCAB	6-carboxycellulose acetate butyrate
CASub	Cellulose acetate suberate
CMCAB	Carboxymethyl cellulose acetate butyrate
CAAdP	Cellulose acetate adipate propionate
GC-MS	Gas chromatography–mass spectrometry
NMR	Nuclear magnetic resonance
UV-Vis	Ultraviolet–visible spectroscopy
HPMCAS	Hydroxypropyl methylcellulose phthalate
CMCS	Carboxymethyl chitosan
MDSC	Modulated differential scanning calorimetry
AFM	Atomic force microscopy
TEM	Transmission electron microscopy
PAA	Polyacrylic acid
HP-β-CD	Hydroxypropyl-β-cyclodextrin
EDS	Energy-dispersive spectroscopy
BET analysis	Brunauer–Emmett–Teller surface-area analysis
PXRD	Powder X-ray diffraction
NIR	Near-infrared spectroscopy
TPS	Terahertz pulsed spectroscopy
ssNMR	Solid-state nuclear magnetic resonance
DVS	Dynamic vapor sorption
IGC	Inverse gas chromatography
XPS	X-ray photoelectron spectroscopy
